# Aberrant binding of mutant HSP47 affects posttranslational modification of type I collagen and leads to osteogenesis imperfecta

**DOI:** 10.1371/journal.pgen.1009339

**Published:** 2021-02-01

**Authors:** Delfien Syx, Yoshihiro Ishikawa, Jan Gebauer, Sergei P. Boudko, Brecht Guillemyn, Tim Van Damme, Sanne D’hondt, Sofie Symoens, Sheela Nampoothiri, Douglas B. Gould, Ulrich Baumann, Hans Peter Bächinger, Fransiska Malfait

**Affiliations:** 1 Center for Medical Genetics, Ghent University Hospital, Ghent, Belgium; 2 Department of Biochemistry and Molecular Biology, Oregon Health & Science University, Portland, Oregon, United States of America; 3 Department of Ophthalmology, UCSF School of Medicine, San Francisco, California, United States of America; 4 Institute of Biochemistry, University of Cologne, Cologne, Germany; 5 Amrita Institute of Medical Sciences and Research Center, Cochin, Kerala, India; 6 Department of Anatomy, Institute for Human Genetics, UCSF School of Medicine, San Francisco, California, United States of America; Murdoch Childrens Research Institute, AUSTRALIA

## Abstract

Heat shock protein 47 (HSP47), encoded by the *SERPINH1* gene, is a molecular chaperone essential for correct folding of collagens. We report a homozygous p.(R222S) substitution in HSP47 in a child with severe osteogenesis imperfecta leading to early demise. p.R222 is a highly conserved residue located within the collagen interacting surface of HSP47. Binding assays show a significantly reduced affinity of HSP47-R222S for type I collagen. This altered interaction leads to posttranslational overmodification of type I procollagen produced by dermal fibroblasts, with increased glycosylation and/or hydroxylation of lysine and proline residues as shown by mass spectrometry. Since we also observed a normal intracellular folding and secretion rate of type I procollagen, this overmodification cannot be explained by prolonged exposure of the procollagen molecules to the modifying hydroxyl- and glycosyltransferases, as is commonly observed in other types of OI. We found significant upregulation of several molecular chaperones and enzymes involved in procollagen modification and folding on Western blot and RT-qPCR. In addition, we showed that an imbalance in binding of HSP47-R222S to unfolded type I collagen chains in a gelatin sepharose pulldown assay results in increased binding of other chaperones and modifying enzymes. The elevated expression and binding of this molecular ensemble to type I procollagen suggests a compensatory mechanism for the aberrant binding of HSP47-R222S, eventually leading to overmodification of type I procollagen chains. Together, these results illustrate the importance of HSP47 for proper posttranslational modification and provide insights into the molecular pathomechanisms of the p.(R222S) alteration in HSP47, which leads to a severe OI phenotype.

## Author summary

Heat shock protein 47 (HSP47) is essential for correct collagen folding. We report a homozygous p.(R222S) substitution in HSP47 in a child with severe osteogenesis imperfecta. The highly conserved p.R222 residue is located within the collagen interacting surface and HSP47-R222S shows a significantly reduced affinity for type I collagen. This altered interaction leads to posttranslational overmodification of type I procollagen. In contrast to other types of OI, this overmodification is not caused by prolonged exposure of procollagen to modifying enzymes, since the intracellular folding rate of type I procollagen appears to be normal. We show significant upregulation of several molecular chaperones and collagen-modifying enzymes and increased binding of several of these molecules to unfolded type I collagen chains upon abnormal HSP47-R222S binding. This suggests a compensatory mechanism for aberrant HSP47-R222S binding, eventually leading to overmodification of type I procollagen chains, and underscores the importance of HSP47 for proper posttranslational modification.

## Introduction

Type I collagen is the most abundant extracellular matrix protein in humans and plays critical roles in many connective tissues such as bone, tendon and skin. The biosynthesis of type I collagen is a particularly complex process, which starts with the synthesis of precursor procollagen chains (pro-α1(I)-chains encoded by *COL1A1* and pro-α2(I)-chains encoded by *COL1A2*). These precursor chains typically consist of a long helical domain, which contains multiple Gly-Xaa-Yaa repeats, flanked by globular carboxy- (C-) and amino- (N-) terminal domains. In the rough endoplasmic reticulum (rER) two pro-α1(I)-chains and one pro-α2(I)-chain associate at their C-propeptide and fold into an elongated triple helix, which propagates in a zipper-like fashion towards the N-terminus. Prior to this helix formation several posttranslational modifications occur, including prolyl 4-hydroxylation, prolyl 3-hydroxylation and lysyl hydroxylation. Galactose or glucosyl-galactose sugars are additionally attached to some of the hydroxyl-lysine residues. The enzymes responsible for these modifications can only act on non-triple helical pro-α-chains, and posttranslational modifications cease once triple helix formation is completed. [1] Correct folding, modification and secretion of the procollagen chains requires the concerted action of over 20 rER-resident molecules including chaperones, enzymes and posttranslational modifiers, collectively coined as the ‘molecular ensemble’. [2] One of these molecules is the molecular chaperone HSP47, which is encoded by the *SERPINH1* gene. HSP47 was discovered in the late 1980s as a 47-kDa collagen-binding and heat shock protein. [3] HSP47 specifically binds to procollagens and has a characteristic expression pattern in cells and tissues that closely correlates with that of collagens. [4,5] It preferentially binds the folded triple helix [6], recognizing Gly-Xaa-Arg triplets across the helix. [7] The elucidation of the HSP47 crystal structure revealed that two HSP47 molecules bind to two of the three α-chains of homotrimeric synthetic collagen model peptides. [8] As such, HSP47 molecules stabilize the triple helix structure during folding and also prevent lateral association of procollagen triple helices in the ER. [9,10] Furthermore, HSP47 acts as an anchor molecule between procollagens and the SH3 domain of TANGO1, a membrane sorting receptor that is present at ER exit sites and that ensures efficient sorting into and enlarging of special procollagen transport vesicles. [11] It has been suggested that HSP47 also accompanies folded procollagen molecules to the cis-Golgi or the ER-Golgi intermediate compartment (ERGIC) in large COPII carriers. [12] In the Golgi apparatus, the pH drop releases HSP47 from procollagen, which is then shuttled back to the ER by its C-terminal RDEL sequence. [13] The importance of HSP47 during development is illustrated by the embryonic lethality of *Serpinh1* knockout mice, which do not survive beyond 11.5 days postcoitus (dpc). Genetic ablation of *Hsp47* results in virtual absence of collagen fibers and disrupted basement membranes in these mouse embryos, which also display abnormally oriented epithelial tissues and ruptured blood vessels. [14] Pathogenic variants in the *SERPINH1* gene (OMIM 600943) cause osteogenesis imperfecta (OI, OMIM 613848) in humans and dogs. The first evidence for the involvement of defective HSP47 in OI came from rough-coated Dachshunds displaying a severe OI phenotype, who were found to harbor a homozygous *SERPINH1* p.(Leu326Pro) variant. [15] In 2010, a homozygous substitution (p.(Leu78Pro)) in HSP47 was described in a young child with severe OI. [16] Subsequently, bi-allelic *SERPINH1* variants were described in seven additional individuals from five unrelated families, suffering from moderately severe to neonatal lethal OI. [17–21] These variants included homozygous or compound heterozygous missense variants (p.(Met237Thr) [17] and p.[(Leu50Arg)];[(Arg405His)] [20]), a homozygous in-frame deletion (p.(Glu105_His108del)) [18], a homozygous frameshift variant (p.(Glu113Valfs*8)) [19], and a compound heterozygous nonsense variant (p.(Asp412*) combined with a >5 kb genomic deletion located 2.37 kb upstream of the transcription start site. [21]

Here, we report the ninth patient suffering from severe OI caused by a novel homozygous *SERPINH1* missense variant (p.(R222S)). We performed in depth characterization of the effect of this p.(R222S) genetic variant in HSP47 in relation to collagen binding, biosynthesis and posttranslational modification.

## Results

### Clinical report

**Fig 1:**
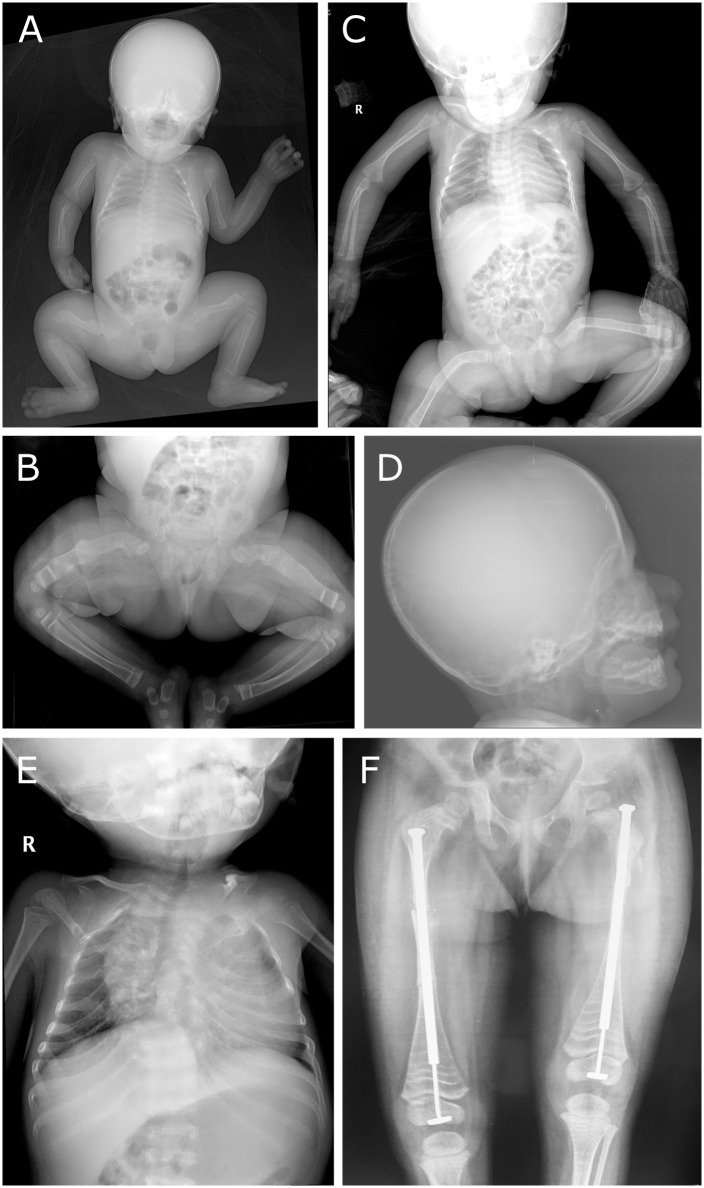
Radiological features. **(A-C)** X-rays at age 4 days **(A)**, 2 months **(B)** and 8 months **(C)** show fractures with callus formation, gracile and thin ribs with fractures, platyspondyly and severe hypomineralization. **(D)** Skull X-ray at age 10 months shows severe thinning and Wormian bones. **(E-F)** X-rays at 3 years of age show crowding of the ribs, scoliosis and femora with ‘Zebra lines’ following regular pamidronate infusions.

The patient is the only child of non-consanguineous, healthy Indian parents. At birth she presented with blue sclerae, a large anterior and posterior fontanel, bilateral bowing of the femur and multiple fractures. A skeletal survey at 4 days of age confirmed fractures with callus formation in both femora, gracile and thin ribs with fractures, platyspondyly and generalized severe hypomineralisation (Fig 1A–1C). Skull X-rays showed severe thinning and Wormian bones (Fig 1D). Intravenous administration of pamidronate was started at the age of 16 days and administered every two months. At the age of 2.5 years she underwent Sheffield telescoping rod insertion for both femurs, after which she started to walk with support. Facial features at this age included a triangular face with relative macrocephaly, a prominent forehead, blue sclerae, micrognatia and dentinogenesis imperfecta. Cardiac examination was unremarkable. Otoacoustic emission test failed bilaterally. At 38 months, her height was 76.5 cm (<1^st^ percentile), weight was 7.8 kg (<1^st^ percentile) and head circumference was 49 cm (>1^st^ percentile). She had a large anterior fontanel, short neck, scoliosis and severe crowding of ribs. X-rays showed good mineralization with ‘Zebra lines’ following regular pamidronate infusions (Fig 1E and 1F). Because the fracture rate was extremely low, the family decided to discontinue pamidronate infusions. At age 4.5 years, she died from pneumonia. No autopsy was performed.

Clinical data of this patient and all reported patients with pathogenic *SERPINH1* variants is summarized in S1 Table.

### Identification of a novel homozygous *SERPINH1* variant

**Fig 2:**
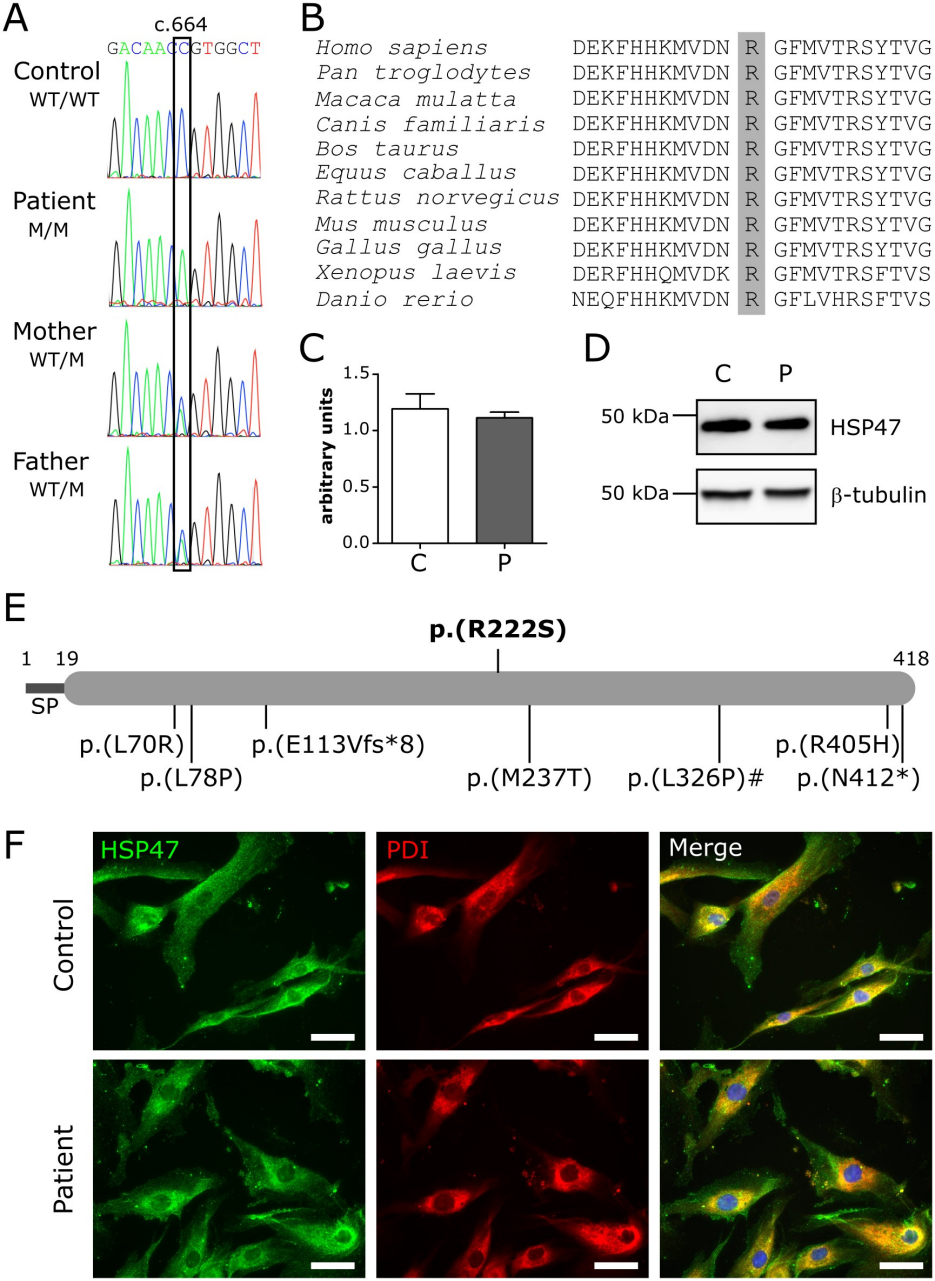
Variant identification and characterization. **(A)** Electropherograms of the identified variant in the patient, unaffected parents and control. WT: wild-type, M: mutant. **(B)** Protein sequence alignment of different species. The affected p.Arg222 residue is shaded in grey. **(C)** RT-qPCR expression analysis for *SERPINH1*. Data are expressed as mean ± SEM. **(D)** Western blot analysis for HSP47. **(E)** HSP47 domain structure with inclusion of all pathogenic variants identified to date. The variant reported here is depicted above the structure. Numbers above the structure indicate amino acid residues. The pathogenic variant with a hashtag (#) depicts the only canine variant identified so far. The reported upstream genomic deletion is not depicted in this image. SP: signal peptide. **(F)** Immunocytochemical analysis showing colocalization of wild-type and R222S HSP47 with the ER-marker PDI. Nuclei are counterstained with DAPI. Scale bar = 50 μm. C: control and P: patient.

After excluding defects in *COL1A1* and *COL1A2*, molecular analysis was performed for all other known genes associated with OI. This revealed a homozygous c.664C>A transversion in the *SERPINH1* gene, resulting in the substitution of a highly conserved amino acid (p.(Arg222Ser) or p.(R222S)) (Fig 2A and 2B). The variant was reported only once in heterozygous state in public databases (1 in 251,474 alleles, queried on November 8, 2020) and the majority (3/4) of *in silico* prediction algorithms considered it to have deleterious effects (S2 Table). According to the guidelines of the American College of Medical Genetics and Genomics, the variant was classified as likely pathogenic (class 4). Segregation analysis revealed that both healthy parents are heterozygous carriers of this variant (Fig 2A). Quantitative reverse transcription (RT-qPCR) showed normal *SERPINH1* mRNA expression in fibroblast cultures derived from the proband, compared to control (Fig 2C). No apparent differences in HSP47 protein levels (Figs 2D and 5) and subcellular localization (Fig 2F) were observed by Western blot and immunofluorescence analysis on dermal fibroblast cultures, indicating that HSP47-R222S protein is stably produced and correctly localized to the ER.

### HSP47-R222S has reduced binding capacity for type I collagen

**Fig 3:**
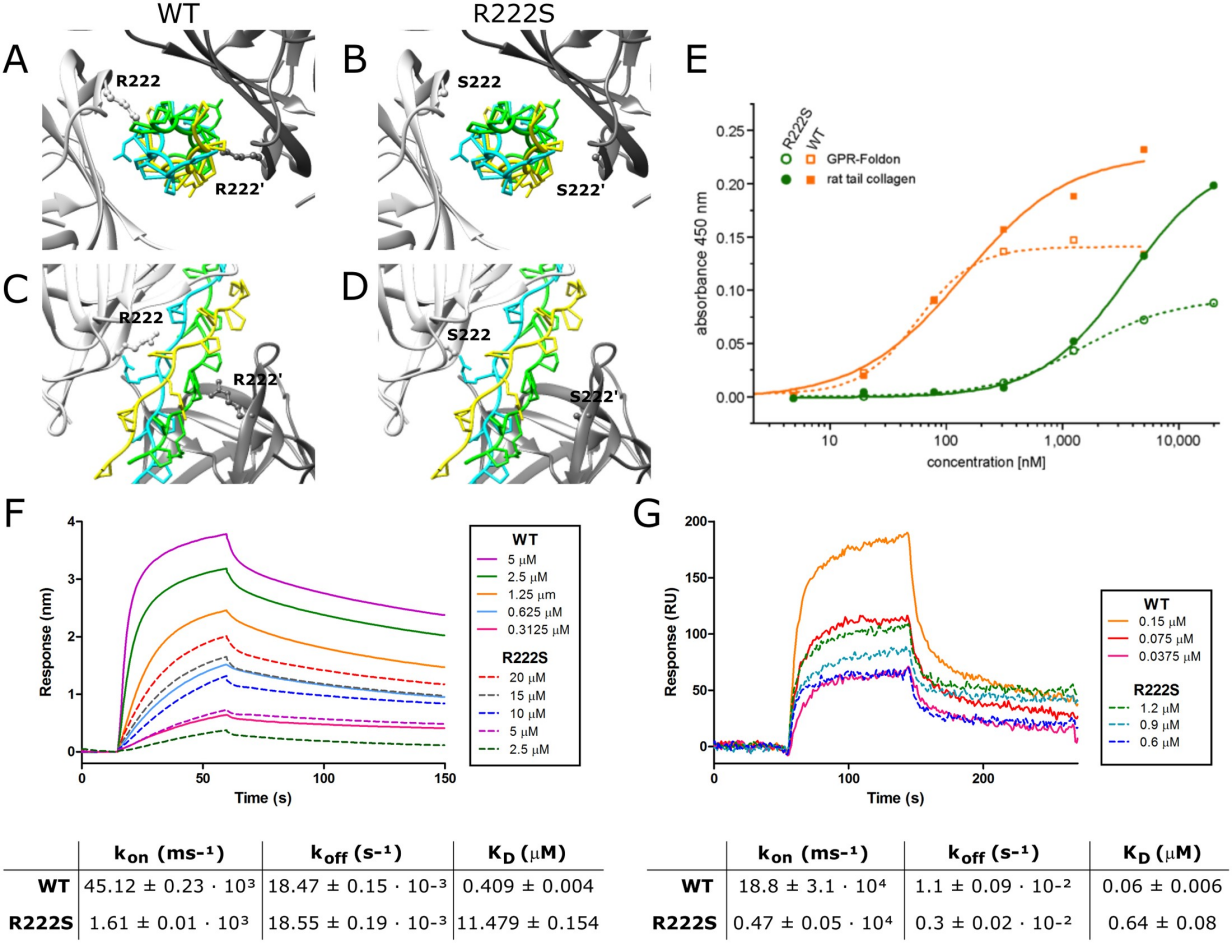
Determination of the binding properties of WT and R222S HSP47. **(A-D)** Crystal structure of the HSP47-collagen interaction with the collagen trimer depicted centrally, surrounded by two HSP47 molecules depicted in light and dark grey. **(A,C)** Wild-type (WT) and **(B,D)** R222S HSP47. **(E)** ELISA binding assay of wild-type (WT) and R222S HSP47 to GPR-foldon and type I collagen from rat tail. **(F-G)** Concentration dependent direct binding of WT and R222S HSP47 proteins with **(F)** GPR-foldon using biolayer interferometry and **(G)** type I collagen using surface plasmon resonance. All curves are averaged by a minimum of three measurements. Tables represent the respective binding affinities. Results are shown as mean ± SD.

Mapping of the substitution on the canine HSP47 crystal structure, which shows 97% sequence identity to the human HSP47, revealed that the affected p.R222 residue is located within the collagen interacting surface of HSP47, suggesting a crucial role for this residue in binding of HSP47 to type I collagen (Fig 3A–3D). [8]

In order to test whether this substitution affected binding of HSP47 to type I collagen, recombinant wild-type and R222S canine HSP47 were produced in *Escherichia coli*. Differential Scanning Fluorimetry showed a similar melting point for both wild-type (54.50°C) and R222S (54.00°C) protein, confirming that the variant does not cause structural instability (S1 Fig).

First, direct binding kinetics of wild-type and R222S HSP47 were analyzed for a collagen-like GPR peptide (GPR-foldon) using Biolayer Interferometry (BLI) and full length pepsinized type I collagen from mouse tail tendon using Surface Plasmon Resonance (SPR) analysis. Wild-type HSP47 was shown to interact in a concentration dependent manner with both immobilized GPR-foldon (Fig 3F) and immobilized type I collagen (Fig 3G). Although HSP47-R222S also showed a concentration-dependent interaction, its ability to bind to the GPR-foldon or type I collagen was markedly reduced, even with higher concentrations of HSP47-R222S protein (Fig 3F and 3G). The equilibrium dissociation constant (K_D_) for wild-type (0.409 ± 0.004 μM) and R222S (11.479 ± 0.154 μM) HSP47 revealed an approximately 25-fold lower affinity of HSP47-R222S for the GPR-foldon with BLI (Fig 3F), while K_D_ values determined by SPR showed a ~10-fold weaker binding affinity of HSP47-R222S (0.64 ± 0.08 μM) to type I collagen compared to wild-type HSP47 (0.06 ± 0.006 μM) (Fig 3G). While the dissociation rate constant (k_off_) was relatively similar between wild-type and R222S HSP47 in both BLI and SPR, the association rate constant (k_on_) was altered, suggesting that HSP47-R222S also shows a slower association with the GPR-foldon (Fig 3F) and type I collagen (Fig 3G).

These findings were subsequently confirmed using an enzyme-linked immunosorbent binding assay (ELISA-like binding assay). Analysis of wild-type HSP47 showed a slightly higher affinity for type I collagen compared to GPR-foldon, with K_D_ values of 139 nM and 56 nM, respectively. For HSP47-R222S a shift in the curves was seen, confirming that the binding of HSP47-R222S to both the GPR-foldon and to full length type I collagen was severely reduced compared to wild-type HSP47. This is reflected by K_D_ values of approximately 1.5 μM (GPR-foldon) and 3.6 μM (type I collagen), which were roughly 25 times worse than for the wild-type HSP47 protein (Fig 3E).

Collectively, these results demonstrate that the affinity of HSP47-R222S for the collagen triple helix is severely reduced, though not completely abolished, in comparison with wild-type HSP47. As such, these findings confirm a significant role of the p.R222 residue for binding of HSP47 to type I collagen.

### HSP47-R222S alters synthesis and stability of type I collagen

**Fig 4:**
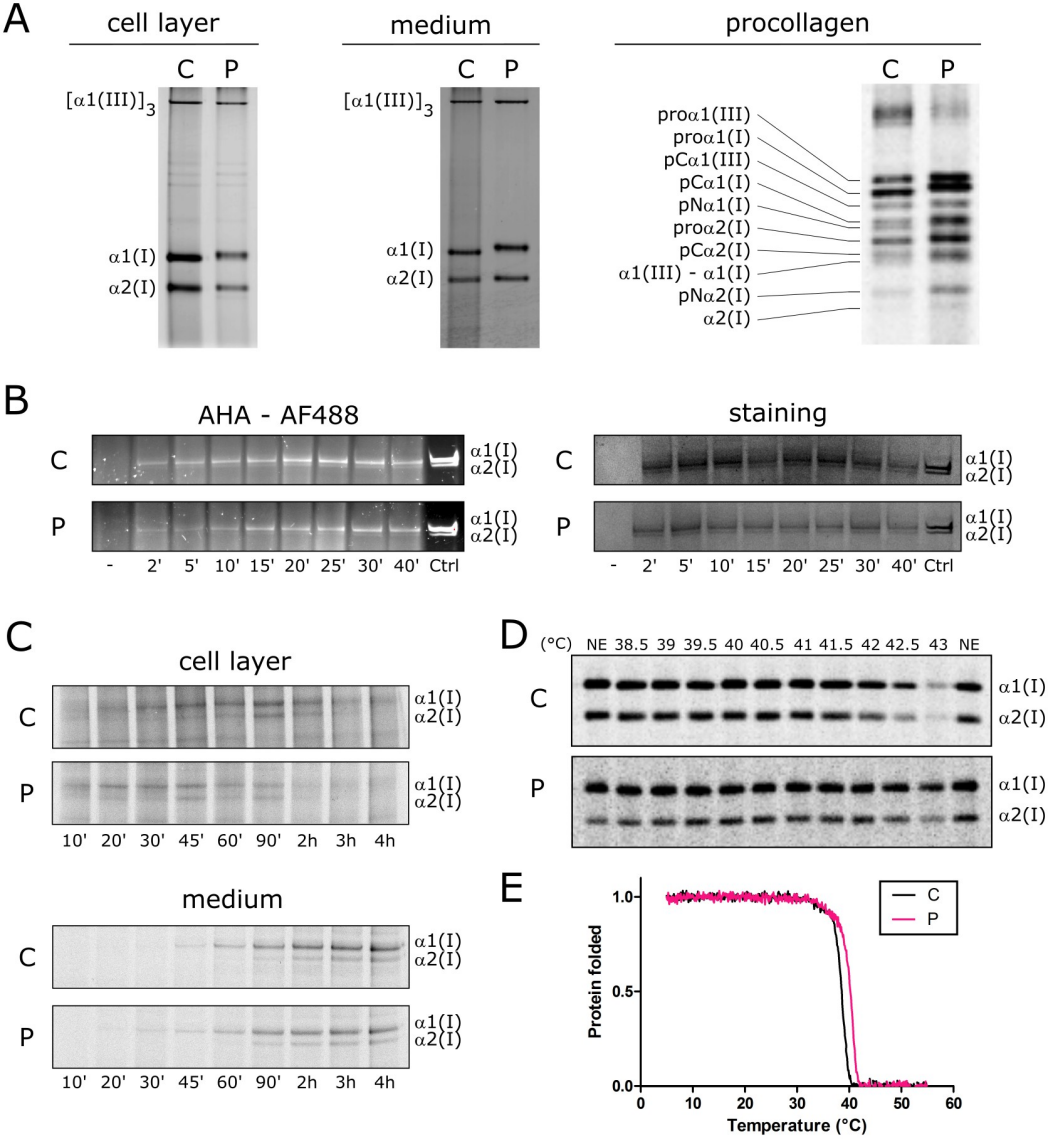
Consequences for type I (pro)collagen synthesis, thermal stability, intracellular folding and secretion. **(A)** Steady-state biochemical (pro)collagen analysis of (pro)collagens harvested from medium and cell layer from dermal fibroblast cultures. **(B)** Intracellular folding assay for type I collagen measured by pulse-chase labeling of type I collagen with AHA-Alexa Fluor 488 and staining of the total amount of type I collagen (with and without AHA) with GelCode Blue Stain Reagent. ‘-‘ denotes Alexa Fluor 488 labeled TBS buffer only, Ctrl: purified AHA incorporated type I collagen from culture medium after overnight chase as a loading control. **(C)** Type I collagen secretion assay. **(D)** Thermal stability of secreted type I collagen molecules with biochemical analysis. **(E)** Melting curve analysis of type I collagen measured in the control (black) and patient (pink) by circular dichroism measurements. C: control, P: patient.

Next, the consequences of the p.(R222S) substitution on HSP47 chaperone functionality and its effects on the biosynthesis and properties of type I (pro)collagen were evaluated. To this end, biochemical analysis of metabolically labeled (pro)collagen chains was performed. Steady-state analysis of pepsinized collagen separated by sodium dodecyl sulfate polyacrylamide gel electrophoresis (SDS-PAGE) showed that the bands representing the type I collagen α-chains showed a decreased electrophoretic mobility in both medium and cell layers of patient fibroblasts compared to control fibroblasts (Fig 4A). These findings are suggestive for posttranslational overmodification of the type I collagen α-chains.

Since HSP47 is required for correct folding of type I procollagen [9], the effect of the p.(R222S) substitution on the folding rate, secretion rate and stability of type I collagen was investigated. A normal intracellular folding (Figs 4B and S2) and secretion rate (Figs 4C and S3) of type I procollagen was detected. In addition, a slightly increased thermal stability of type I collagen α-chains (Fig 4D) was observed in patients’ fibroblast cells compared to control, which is most likely a consequence of overmodification. This was confirmed using circular dichroism (CD) measurements, which showed a slight shift to higher temperatures in the melting curve of type I collagen in the patient sample compared to control both at a heating rate of 0.1°C/min (Fig 4E) and 1°C/min (S4 Fig).

Overall, these findings show that the reduced binding of HSP47-R222S to type I procollagen chains is associated with type I (pro)collagen overmodification, which leads to a mildly increased thermal stability. Despite the observed overmodification, folding and secretion of type I collagen was normal.

### HSP47-R222S increases posttranslational modification of type I collagen

In order to quantitate and characterize the posttranslational modifications of type I collagen, amino acid analysis and mass spectrometry was performed to determine the modification on selected residues in the triple helical region.

Overall, amino acid analysis revealed a significant increase in 3-hydroxylated proline and 4-hydroxylated proline levels, with an increase of 65% and 2.5%, respectively, in the patient sample compared to control (S5A, S5D and S5E Fig). The relatively low increase in 4-hydroxylated proline can be attributed to the fact that proline residues in the Yaa position are typically almost fully 4-hydroxylated. Whereas mass spectrometry analysis showed that the A1 site (Pro-986) of the α1(I)-collagen chain was almost completely 3-hydroxylated in both patient and control dermal fibroblasts (S6A Fig), 3-hydroxylation for the A3 site (Pro-707) was increased in patient compared to control (S6B Fig).

In addition, amino acid analysis demonstrated that type I collagen produced by patient cells contained 60% more hydroxylysine moieties compared to control (S5B, S5D and S5E Fig). This is substantially higher when compared to the initially reported patients with *CRTAP* and *LEPRE1* mutations, where an increase in hydroxylysines is seen ranging between 31.3–35.1% and 32.4–35.3%, respectively. [22,23] Mass spectrometry confirmed that Lys-756 of the α1(I)-chain showed a markedly increased amount of hydroxylysine in the patient sample compared to control (S6E Fig). Hydroxylysine residues are important for crosslinking, but can also be glycosylated. [24] Type I collagen from patient fibroblasts harbored about 50% more glucosyl-galactosyl-hydroxylysines compared to control (S5C Fig). Lys-174 of the α1(I)-chain was nearly completely modified to glucosyl-galactosyl-hydroxylysine in the patient (S6C Fig), and increased amounts of galactosyl hydroxylysine and glucosyl-galactosyl-hydroxylysine were also found at Lys-531 of the α1(I)-chain compared to control (S6D Fig). These results confirmed the overmodification of type I collagen seen on biochemical analysis (SDS-PAGE).

### HSP47-R222S leads to upregulation of modifying enzymes and chaperones as well as alternative composition of the molecular ensemble

**Fig 5:**
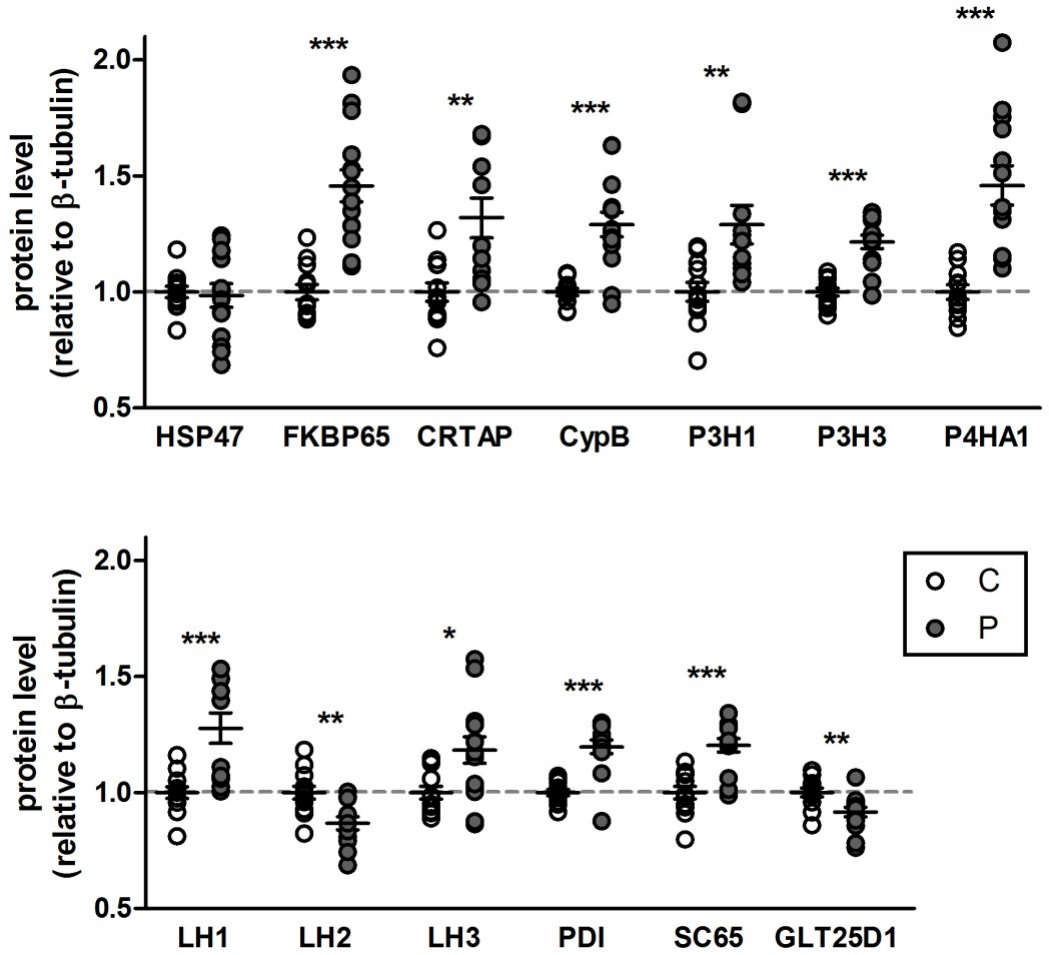
Comparison of protein levels of the molecular ensemble. Semi-quantitative Western blotting was performed to see effects of the *SERPINH1* variant on actual protein levels of the molecular ensemble involved in collagen biosynthesis. The protein signals were normalized to β-tubulin levels and each value was set to 1 for the control. Data presented are means ± SEM and individual data points represent independently prepared cell lysates (n>10). *: p<0.05, **: p<0.01 and ***: p<0.001 by unpaired t test. C: control, P: patient.

In an attempt to explain the observed overmodification of type I collagen that was unexpectedly combined with normal folding, the protein and mRNA expression levels of various enzymes and chaperones involved in procollagen biosynthesis were quantified by Western blot and RT-qPCR analysis, respectively, in patient and control fibroblasts. Western blot analysis indicated a significant upregulation for the majority of the evaluated proteins, including the peptidyl-prolyl cis/trans isomerase (PPIase) FK506 binding protein 65kDa (FKBP65, encoded by *FKBP10*) and cyclophilin B (CypB, encoded by *PPIB*); posttranslational modifiers, prolyl 3-hydroxylase 1 (P3H1, encoded by *LEPRE1* (*P3H1*)), prolyl 3-hydroxylase 3 (P3H3, encoded by *P3H3*), prolyl 4-hydroxylase 1 (P4HA1, encoded by *P4HA1*), lysine hydroxylase 1 (LH1 encoded by *PLOD1*) and 3 (LH3, encoded by *PLOD3*); and posttranslational modifier binding proteins protein disulfide isomerase (PDI, encoded by *P4HB*), cartilage associated protein (CRTAP, encoded by *CRTAP*) and synaptonemal complex 65 (SC65 or P3H4, encoded by *P3H4*). The levels of the telopeptide-specific lysine hydroxylase 2 (LH2, encoded by *PLOD2*) and glycosyltransferase 25 domain-containing 1 (GLT25D1, encoded by *COLGALT1*) were decreased, whereas HSP47-RSSS2 protein levels remained unaltered compared to control (confirming our previous findings) (Figs 5 and S7).

To evaluate whether the increased protein levels resulted from upregulated gene expression, RT-qPCR was performed to check the corresponding mRNA expression levels. For the genes encoding proteins that were upregulated, expression analysis revealed either a significant increase in transcript levels (*FKBP10*, *PPIB*, *P3H1*, *P3H3*, *PLOD1*, *PLOD3* and *P4HB*) or a trend towards increased expression (*CRTAP*, *P4HA1* and *P3H4*). Expression levels of *PLOD2* were not significantly different, but surprisingly, *COLGALT1* was upregulated at transcriptional level, despite decreased protein levels (S8 Fig).

**Fig 6:**
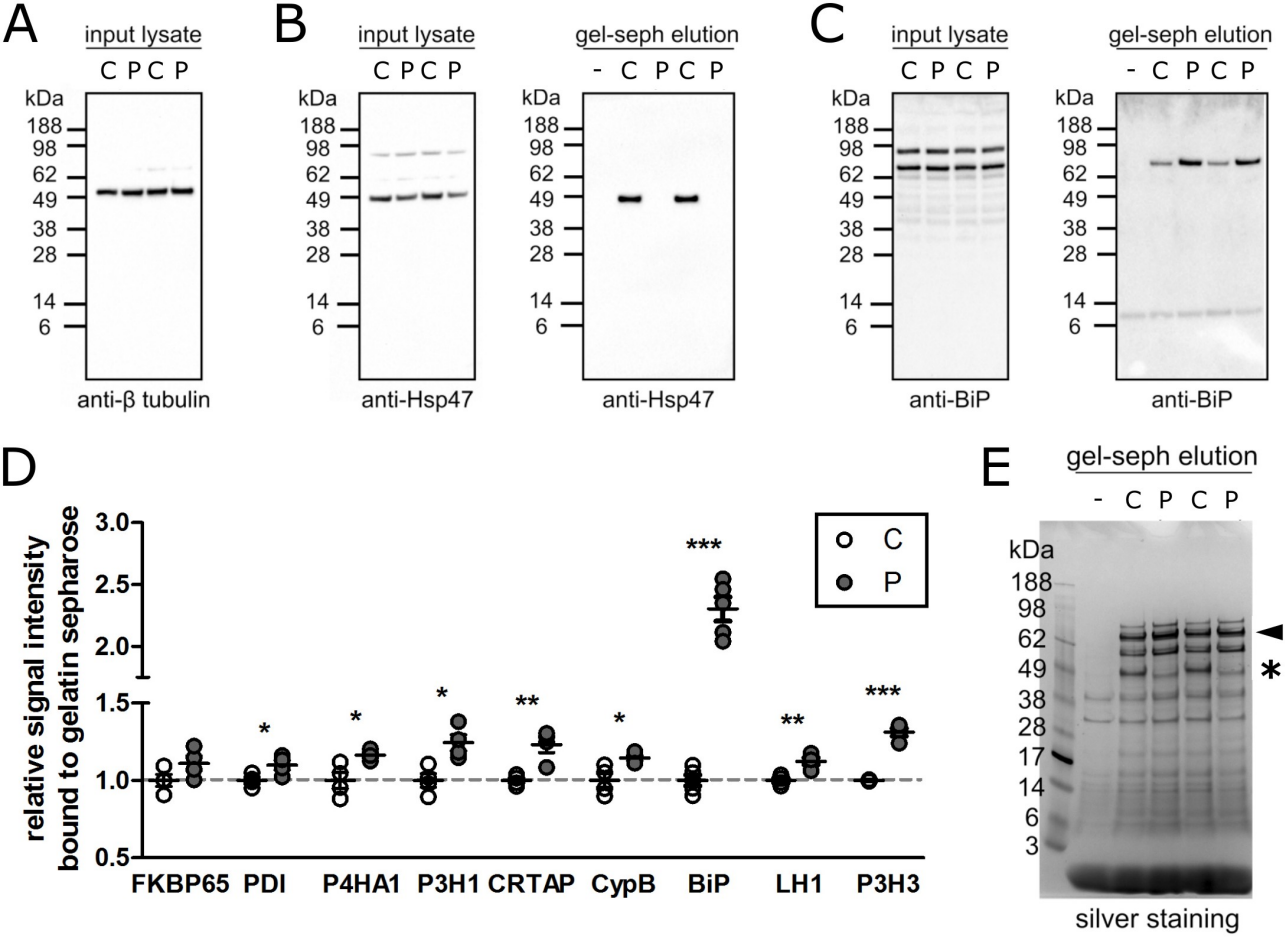
Identification of proteins interacting with gelatin sepharose. Proteins that bound tightly to gelatin sepharose were eluted with SDS sample buffer. **(A-C)** Images of Western blots are shown. Cell lysate and gelatin sepharose (gel-seph) elution represent the input cell lysate that was loaded onto gelatin sepharose and the eluted fractions from gelatin sepharose. The blot against β-tubulin is an input control. The blots with clear differences between WT and R222S HSP47 are shown. Blots against the other proteins are shown in S9A Fig. **(D)** Semi-quantitative Western blotting was performed for proteins that were reported to interact with gelatin sepharose. The protein signals of the control sample were set to 1 and compared to the patient sample. Data presented are means ± SEM and individual data points represent four or more independently prepared cell lysate (n>4). *: p<0.05, **: p<0.01 and ***: p<0.001 by unpaired t test. **(E)** Silver staining was performed for the eluted fractions from the gelatin sepharose. The arrowhead and asterisk (*) highlight the predicted migration of BiP and HSP47, respectively. ‘-‘ indicates the eluted fraction from gelatin sepharose mixed with TBS buffer instead of cell lysate as a blank.

We next performed a gelatin sepharose pulldown assay to evaluate binding of wild-type and HSP47-R222S as well as the various biosynthetic enzymes and chaperones to denatured type I collagen. Wild-type, but not R222S, HSP47 was able to interact with denatured collagen (Fig 6A, 6B and 6E). In contrast, CRTAP, CypB, P3H1, P3H3, P4HA1, LH1, PDI and BiP showed a significantly increased binding to gelatin sepharose in the absence of HSP47 binding (Figs 6D and S9A), with BiP demonstrating the highest increase compared to control fibroblasts (Fig 6C–6E), despite unaltered endogenous expression and protein levels (Figs 6C, S8 and S10A). Binding to gelatin sepharose was not significantly increased for FKBP65 and no binding was observed for GRP94 (HSP90), Calreticulin and LH3 (S9B Fig).

These findings suggest that the molecular ensemble of chaperones, enzymes and modifiers tries to compensate for the abnormal HSP47 binding, presumably by occupying the vacant HSP47 binding sites, eventually leading to overmodification without affecting folding and secretion.

### HSP47-R222S does not induce classical ER stress or autophagy responses

Finally, we evaluated if the identified *SERPINH1* variant induced an unfolded protein response and/or signs of increased autophagic elimination in patient fibroblasts. To test for the activation of different branches of ER stress pathways, levels of BiP, XBP1-spliced, CHOP, phospho-eIF2α and ATF6, which are induced upon accumulation of unfolded proteins, were analyzed by Western blot. Protein levels of the general ER chaperone BiP (S10A Fig), ATF6 (S10B Fig) and phospho-eIF2α (S10C Fig) remained unaltered in both patient and control fibroblasts. Endogenous levels of the ER stress marker XBP1-spliced and the proapoptotic transcription factor CHOP could not be detected, but fibroblasts did not respond differently to tunicamycin (Tu)- or thapsigargin (Th)-induced ER stress (S10A Fig). The amount of the endogenous autophagy marker membrane-bound LC3B-II, which is incorporated into autophagosomes, did not differ between the patient and control (S10D Fig).

## Discussion

**Fig 7:**
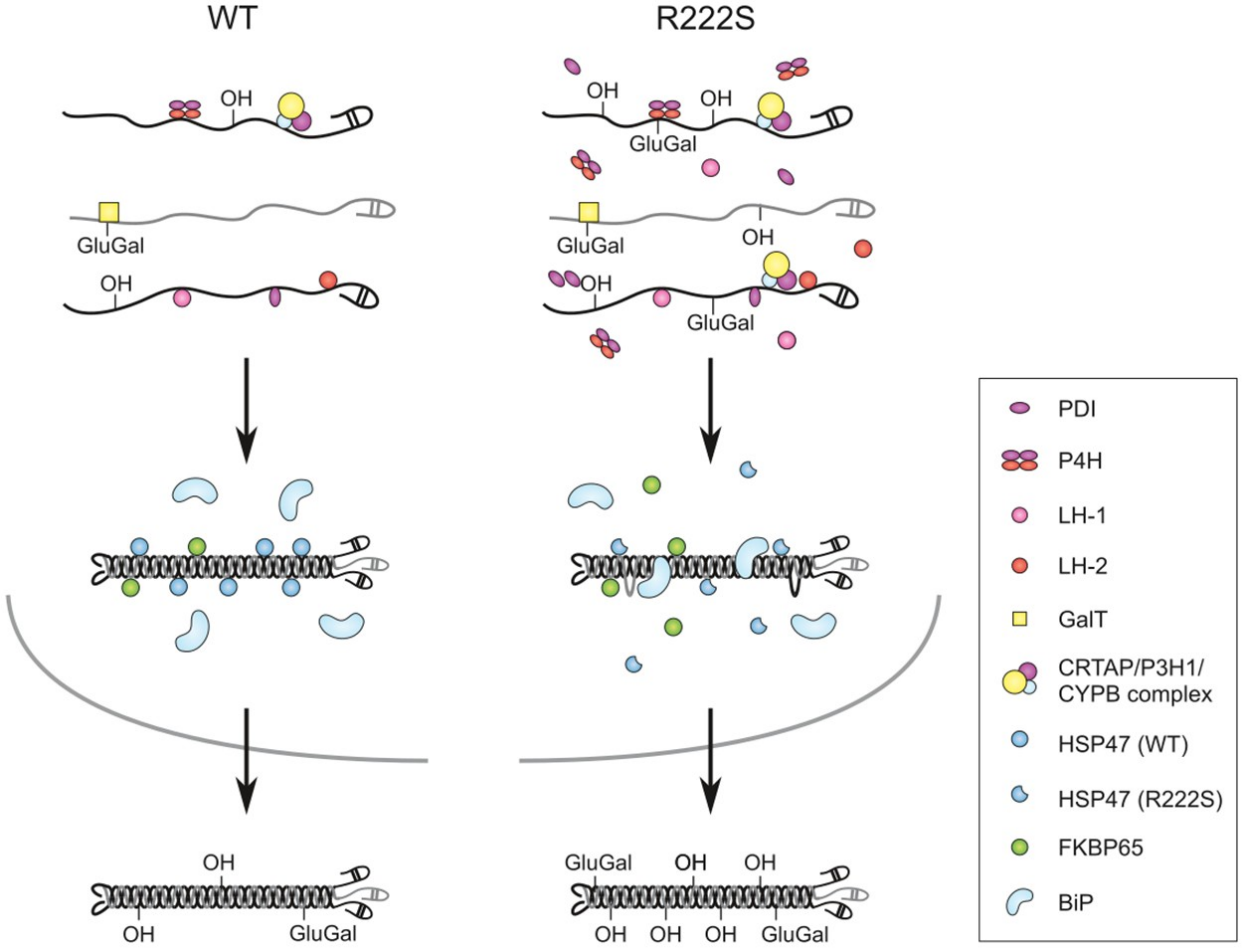
Proposed mechanism of HSP47-R222S. Wild-type (WT) is depicted on the left, R222S on the right. In R222S fibroblasts, increased levels of modifying enzymes are detected, which can bind the nascent procollagen α1(I)- and α2(I)-chains (depicted in black and grey, respectively) and eventually result in overmodification of the respective pro-α(I)-chains due to an increased enzyme to α-chain ratio. Following trimerisation of the pro-α-chains, HSP47-R222S binds with lower affinity to the procollagen triple helix. The lack of triple helix stabilization by defective HSP47 binding potentially result in local unwinding of the triple helix, thereby making the individual pro-α-chains accessible to the modifying enzymes for additional posttranslational modifications. In addition, the vacant HSP47 binding sites can presumably be occupied by other chaperones (*e*.*g*. BiP). This will result in overmodification of pro-α-chains without delaying the folding or secretion of type I procollagen. The overcompensation by the molecular ensemble in combination with the fact that HSP47-R222S can still bind to type I procollagen, allows it to still function as a guide molecule, bind to TANGO1 and direct procollagen molecules to special secretory vesicles, resulting in secretion of overmodified type I procollagen. The incorporation of overmodified type I collagen will subsequently result in aberrant collagen fibrillogenesis.

We report a detailed pathomechanistic characterization of a novel homozygous missense variant, p.(Arg222Ser) in the HSP47 encoding gene (*SERPINH1)* which was identified in a child with a severe form of OI. HSP47 is a chaperone essential for efficient folding of collagen in the ER. We show that the substitution of the highly conserved p.Arg222 residue, which is located in the collagen interacting surface of HSP47, by a serine residue results in reduced binding of HSP47 to type I collagen. This pathogenic *SERPINH1* variant leads to posttranslational overmodification of type I procollagen chains, likely resulting from overcompensation by other chaperones and molecules involved in collagen folding and biosynthesis, as demonstrated by their significant upregulation in patients’ dermal fibroblasts, and increased binding to (denatured) type I collagen. A proposed mechanism is depicted in Fig 7.

We compared our findings with those observed in all hitherto reported pathogenic human (n = 7) and canine (n = 1) *SERPINH1* variants and in the *Serpinh1* knock-out (*Serpinh1*^*-/-*^) mice (available data is summarized in Table 1 and missense variants are mapped to the HSP47 crystal structure in S11 Fig) and discuss their implications for type I procollagen biosynthesis and bone formation in the context of understanding the pathomechanisms underlying the observed OI phenotypes.

**Table 1:** Summary of the biochemical consequences of reported HSP47 defects.

	Human				Dog	Mouse
	p.[(Arg222Ser)]; [(Arg222Ser)]	p.[(Leu78Pro)]; [(Leu78Pro)]	p.[(Met237Thr)]; [(Met237Thr)]	p.[(Asp412*)], [p.?]	p.[(Leu326Pro)]; [(Leu326Pro)]	knockout
**Reference**	Present report	Christiansen et al.	Duran et al.	Schwarze et al.	Drögemüller et al., Lindert et al.	Ishida et al., Nagai et al.
**Phenotype**	Severe OI	Severe OI	Moderately severe OI	Moderate OI	OI	embryonic lethal
**HSP47 protein instability**	No	Yes	Yes	Yes	decreased (50%)	/
**HSP47 localization**	ER	absent	ER-related vesicles	absent	ER	/
**Type I collagen migration (SDS-PAGE)**	Delayed	Normal	Normal	Normal	Delayed	Delayed ^a^
**COL1 overmodification**	Yes	No	ND ^b^	No	Yes	ND
**3-hydroxylation of Pro-986**	Normal	Normal	ND	ND	Normal	ND
**Accumulation of COL1**	ND	Golgi	ER-related vesicles	Golgi (slightly)	ER	ER
**ER dilatation**	ND	ND	ND	ND	Yes	Yes
**ER stress markers**	Normal	ND	ND	ND	upregulated	upregulated
**Autophagy marker**	Normal	ND	ND	ND	Normal	upregulated
**COL1 secretion**	Normal	Slow	ND	ND	Slow	Slow
**COL1 thermal stability**	Increased	Increased	ND	ND	ND	ND
**Inefficient COL1 processing**	No	ND	ND	ND	Yes	Yes
**Bone crosslinking alterations**	ND	ND	ND	ND	Yes	ND

Reported pathogenic variants that were not further explored experimentally were not included in this table.

^a^ in the presence of ascorbate.

^b^ ND: not determined.

We show that HSP47 proteins harboring the p.(Arg222Ser) variant are stably expressed and correctly localized to the ER. As such, the phenotype is not caused by reduced availability or mislocalization of HSP47. Reduced amounts of HSP47 were nonetheless observed for the p.(Met237Thr), p.(Leu326Pro) and p.[(Asp412*)];[p.?] variants. [15–17,21,25] For p.(Leu78Pro), HSP47 was shown to be unstable and completely degraded via the proteasome. [16] Immunofluorescent staining showed normal HSP47 localization to the ER in p.(Leu326Pro) cells [15,25], while in p.(Met237Thr) cells, a punctate pattern was observed, with accumulation of HSP47 in vacuolar-like compartments at the periphery of the cell. [17] The fact that on the one hand, HSP47 levels were not reduced in our OI patient, and that on the other hand *Serpinh1*^*+/-*^ mice were phenotypically normal, despite marked decrease of HSP47 [14], underscores the notion that partially decreased levels of HSP47 alone are not solely responsible for the observed OI phenotype.

HSP47 binding prevents local or reverse unfolding of the procollagen α-chains during triple helix formation, and prevents procollagen aggregate formation, which facilitates the transport of procollagen to cis-Golgi. [9,10] Several arginine-containing HSP47-binding motifs with high, medium and low binding affinity have been suggested on the surface of the procollagen triple helix of type I and other collagens. [7,26,27] An important role for p.Arg222 in the binding of HSP47 to type I collagen was suggested by the co-crystal structure of canine HSP47 and a homotrimeric collagen model peptide. [8] Using independent techniques, we confirmed that the binding affinity of HSP47-R222S for type I collagen is severely diminished, although not completely abolished. These observations indicate that the OI phenotype in our patient results from a diminished binding of HSP47-R222S to type I procollagen, rather than from reduced HSP47 availability. Diminished type I collagen binding affinity was recently also demonstrated for the previously reported p.(Leu78Pro) and p.(Leu326Pro) HSP47 variants. [28]

It is well established that monoallelic glycine substitutions and other structural defects in *COL1A1* and *COL1A2* and bi-allellic pathogenic variants in *CRTAP*, *LEPRE1* (*P3H1*) and *PPIB* delay procollagen folding, leading to prolonged exposure of the nascent α-chains to hydroxyl- and glycosyltransferases in the ER. [29,30] SDS-PAGE analysis on cultured fibroblasts of patients with these defects typically shows a slower migration pattern of overmodified α1(I)- and α2(I)-chains. We also observed clear overmodification of the type I (pro)collagen chains on SDS-PAGE, a finding which was confirmed by amino acid analysis and mass spectrometry data, which showed markedly increased hydroxylation and glycosylation of proline and lysine residues. This is also in line with the increased thermal stability of type I collagen observed in our patients’ fibroblasts. Despite increased posttranslational overmodification, procollagen folding was not significantly delayed in patient fibroblasts. This suggests that the overmodification could not be explained by prolonged exposure of the pro-α-chains to hydroxyl- and glycosyltransferases. SDS-PAGE of dermal collagen and mass spectrometry analysis of bone collagen also revealed increased modifications for the canine p.(Leu326Pro) variant, including increased hydroxylation of non-crosslinking and crosslinking lysines. [25] In addition, abnormalities in bone crosslinking were observed for the latter substitution. The authors suggested that this could negatively influence trabecular bone formation, thus contributing to the OI pathology. Crosslinking studies were not performed in our patient, nor in the other reported variants. Biochemical analysis did not reveal signs of overmodification in p.(Leu78Pro), p.(Met237Thr) and p.[(Asp412*)];[p.?] fibroblasts. [16,17,21] The discrepancy between these observations remains unexplained but could result from variant-specific (compensatory) effects, or, alternatively, the overmodifications caused by the latter variants could be too mild to be picked up in fibroblast culture.

In an attempt to explain the observed overmodification, we analyzed the expression and protein levels of several chaperones and enzymes involved in folding and posttranslational modification of procollagen in the patients’ dermal fibroblasts. Our Western blot results show that aberrant binding of HSP47 to type I procollagen results in increased levels of FKBP65, PDI, P4HA1, CypB, P3H1, CRTAP, P3H3, SC65, LH1 and LH3. In a gelatin sepharose pulldown assay, we also observed a significantly increased binding capacity to denatured type I collagen for several of these molecules (PDI, P4HA1, P3H1, CRTAP, CypB, LH1, but also BiP). The absence of increased binding of FKBP65, despite increased protein levels, can be explained by the weaker binding affinity between FKBP65 and collagen compared to FKBP65 and HSP47. [31] Since mutant HSP47 levels are not altered in our patient, FKBP65 preferentially binds to HSP47.

These findings suggest that the observed overmodification of type I (pro)collagen in our patient can be the result of an increased binding capability of this ‘molecular ensemble’ to type I procollagen. Increased levels of modifying enzymes can lead to overmodification due to an increased enzyme to α-chain ratio. In addition, the lack of triple helix stabilization by defective HSP47 binding potentially results in local unwinding of the triple helix, thereby making the individual pro-α-chains accessible to the modifying enzymes for additional posttranslational modifications. Furthermore, vacant HSP47 binding sites can presumably be occupied by other chaperones (Fig 7). Together, these events lead to overcompensation by the molecular ensemble, which, to the best of our knowledge, has not yet been described as a cause of overmodification in pathogenesis of OI. Our findings potentially suggest a role for HSP47 as a regulator of posttranslational modification. Interestingly, previous studies have indicated a role for HSP47 as a negative regulator of prolyl-4-hydroxylation [32] and LH2 [33]. Duran et al. showed that HSP47 forms a chaperone complex with FKBP65 and BiP that regulates the occupancy and/or the activity of LH2 on telopeptide lysine residues, in which LH2 activity is positively regulated by FKBP65 and negatively regulated by HSP47. Fibroblasts from an OI patient harboring the p.(Met237Thr) substitution showed increased LH2 levels, associated with a significant increase in telopeptide lysyl hydroxylation. [33] In contrast, we observed decreased levels of LH2, but we did not assess telopeptide modifications. The discrepancy in LH2 expression levels between our findings and those by Duran et al. possibly reflects variant-specific outcomes. It remains possible that the HSP47-FKBP65-BiP complex contains other ER resident chaperones and molecules, each of which can be affected when HSP47 is defective.

In line with normal folding and secretion of procollagen, we did not observe upregulation of ER stress markers (BiP, CHOP, XBP1-spliced, phospo-eIF2α or ATF6) in fibroblasts from our patient, nor did we observe induction of autophagy. As such, classical ER stress and/or autophagy pathways were not activated and most likely do not contribute to the phenotype of our patient. In contrast, in canine p.(Leu326Pro) fibroblasts, secretion of type I and type V collagen was slightly decreased and type I procollagen accumulated in the ER, leading to activation of ER stress response markers BiP and phospho-eIF2α and dilatation of ER cisternae. [25] Accumulation of type I procollagen in the ER and enlarged ER cisternae were also observed in fibroblasts from *Serpinh1*^-/-^ mice. *Serpinh1*^-/-^ mouse embryonic fibroblasts showed induction of CHOP and splicing of XBP1 [34], and involvement of the autophagy-lysosomal pathway to clear misfolded trimeric collagen aggregates. [35] These observations suggest that, in contrast to our findings, the *Serpinh1*^-/-^ mouse and the canine p.(Leu326Pro) HSP47 show partial intracellular retention of procollagen with activation of an ER stress response potential having a causative role in the OI pathomechanism. [25,34,36]

In summary, our study confirms that bi-allelic *SERPINH1* variants result in severe OI and illustrates that the p.(R222S) variant compromises the interaction between HSP47 and type I procollagen, which disturbs the HSP47-mediated quality control of collagen biosynthesis and triggers a cascade of molecular events resulting in altered type I collagen modification and stability. Although the role of overmodification in OI pathology remains unclear, we hypothesize that the secretion of overmodified type I collagen can potentially hamper lateral fusion of collagens, which can severely compromise collagen fibrillogenesis. In addition, defective crosslinking could potentially impair osteoblast maturation and increase osteoclast recruitment, which can affect bone mineralization. [37] Therefore, the incorporation of overmodified type I collagen likely has a detrimental effect on collagen fibrillogenesis, bone ECM and cell populations, thereby affecting cell-matrix interactions and signaling, and eventually contributing to the bone fragility observed in our patient. Nevertheless, the clinical severity of classical OI does not correlate with the extent of posttranslational overmodification, thereby indicating that the role of overmodification in OI pathology is complex. Since patients with HSP47 defects typically present with an OI phenotype, bone is the main tissue for which manifestations are documented. Of note, some patients with HSP47 defects present with joint hypermobility and generalized hypotonia, suggesting that also tendon/ligaments and muscle function can be compromised. In addition, respiratory problems are observed in 5 out of 9 reported patients, including our patient (S1 Table). Since only a small number of patients with HSP47 defects has been reported, it is hard to draw conclusions on extraskeletal manifestations. The observation that bone is the most prominently affected tissue when HSP47 is defective, despite the abundant expression of type I collagen in multiple tissues and the ability of HSP47 to chaperone several types of collagen, is currently not well understood. There could be several explanations. First, differences are observed in the number and affinity of HSP47-binding motifs for different collagen types [7]. This indicates that not all collagen types are dependent on HSP47 in the same way. Since the α1- and α2-chain of type I collagen harbor the highest number of HSP47 binding sites, this possibly explains why type I collagen is most affected. Second, the expression of HSP47 might differ between different tissues and cell types, which can also add to the final (tissue-specific) effect. Third, posttranslational modifications differ between tissues. For example, the amount of O-glycosylation was observed to be lower in bone compared to skin and tendon. [2,38] It appears that bone is more sensitive to overmodification since small changes can have a huge effect. Therefore, even subtle alterations in posttranslational modification of collagen might have more devastating effects in bone compared to other tissues. Of note, as we used dermal fibroblasts for our experiments because of the unavailability of osteoblasts, we cannot exclude tissue-specific differences.

Comparison of our results with those obtained in other reported pathogenic human/canine *SERPINH1* variants reveals variant-specific outcomes both on HSP47 stability and subcellular localization and on its collagen chaperoning function, leading to different functional consequences on type I procollagen, and different clinical outcomes. As such, the observed clinical and biochemical differences most likely reflect a combination of the location and nature of the substitution in the HSP47 structure, the resulting protein stability of mutant HSP47 and its subcellular localization, the remaining binding affinity of mutant HSP47 to procollagen and the ability of mutant HSP47 to interact with and/or regulate other proteins, affecting downstream pathways to a different extent. Although the precise mechanisms that drive bone fragility in these patients remain poorly understood, the observed alterations likely interfere with proper collagen fibril assembly in the ECM and as such disturbed bone formation.

## Materials and methods

### Ethics statement

This study was approved by the Ethics Committee of Ghent University Hospital, Belgium (number 2013/1118). Written informed consent was obtained from the parents of the affected child.

### Patient information and material

A skin biopsy was taken from the patient and blood samples were obtained from both parents. Genomic DNA was extracted using the Gentra Puregene extraction method (Qiagen, Hilden, Germany).

### Molecular analysis

All coding exons and flanking intronic regions of OI-associated genes (*COL1A1*, *COL1A2*, *IFITM5*, *LEPRE1*, *CRTAP*, *PPIB*, *FKBP10*, *PLOD2*, *SERPINH1*, *SERPINF1*, *BMP1*, *TMEM38B*, *WNT1*, *SP7* and *OASIS*) were PCR amplified and subsequently pooled, indexed and subjected to Nextera XT library preparation (Illumina, San Diego, CA, USA) prior to sequencing on the MiSeq instrument (Illumina). Data were mapped and variants were called using the CLC Genomics Workbench (Qiagen) and subsequently annotated and analysed. [39] Confirmation and segregation analysis was performed by PCR amplification of the corresponding exon of *SERPINH1* (NM_001235.3) followed by bidirectional sequencing using the BigDye Terminator Cycle Sequencing kit and the ABI 3730XL DNA Analyzer (Applied Biosystems, Foster City, CA, USA). The pathogenicity of the variant was evaluated using the Alamut Visual software (version 2.3, Interactive Biosoftware, Rouen, France) including the prediction tools SIFT, PolyPhen2, AlignGVGD and MutationTaster and its occurrence was assessed using the Genome Aggregation Database (gnomAD release 2.0.2) (http://gnomad.broadinstitute.org) and the Osteogenesis Imperfecta variant database (http://oi.gene.le.ac.uk/home.php?select_db=SERPINH1). The variant was classified according to the standards and guidelines of the American College of Medical Genetics and Genomics. [40] The canine HSP47 crystal structure was retrieved from the RCSB Protein Data Bank (www.rcsb.org, accession number 4AU3) and used for variant modelling using the UCSF Chimera software (version 1.10, build 40293).

### Expression analysis

Primary dermal fibroblast cultures were established from a skin biopsy and grown and maintained in Dulbecco’s Modified Eagle Medium (DMEM) (Gibco, Thermo Fisher Scientific, Waltham, MA, USA) supplemented with 10% fetal bovine serum Good (Pan Biotech, Aidenbach, Germany), 1% non-essential amino acids (Gibco), 1% penicillin/streptomycin (Gibco), 1% kanamycin (Gibco) and 0.1% amphotericin B (Gibco) and incubated at 37°C with 5% CO_2_. Total RNA was extracted from fibroblast cultures in triplicate using the RNeasy kit (Qiagen) with on-column DNase digestion, followed by cDNA synthesis using the iScript cDNA Synthesis Kit (Bio-Rad Laboratories, Hercules, CA, USA) and RT-qPCR for *SERPINH1* and genes encoding proteins involved in collagen biosynthesis (primers available upon request) was performed in duplicate. Data analysis was performed with the qBasePlus software (Biogazelle, Ghent, Belgium) using reference genes *HPRT1*, *YWHAZ* and *RPL13A* for normalization.

### Immunoblotting

Dermal fibroblasts from the patient and a healthy control were grown until confluency, incubated for 4 hours in either the absence or presence of the ER stress inducers tunicamycin (10 μg/ml) or thapsigargin (1 μM) (both Sigma-Aldrich, St. Louis, MO, USA) and were subsequently lysed with RIPA buffer (Sigma-Aldrich) containing protease (Roche, Basel, Switzerland) and phosphatase inhibitors (Sigma-Aldrich). An aliquot was subjected to SDS-PAGE and transferred to nitrocellulose membranes, immunolabeled with primary antibodies against HSP47 (ab54874, Abcam, Cambridge, United Kingdom), BiP (#3183, Cell Signaling Technologies (CST), Danvers, MA, USA), CHOP (#2895, CST), XBP1-spliced (647502, Biolegend, San Diego, CA, USA) or LC3B (L7543, Sigma-Aldrich) and horseradish peroxidase-conjugated secondary antibody (CST), and detected with chemiluminescence. For loading control, membranes were stripped and reprobed with a β-tubulin antibody (ab6046, Abcam).

### Immunofluorescence

Dermal fibroblasts were seeded at 3×10^5^ cells/well in eight-well Lab-Tek chamber slides (Thermo Scientific, Waltham, MA, USA) and grown under standard conditions as described above. At confluency, cells were fixed with 4% (w/v) paraformaldehyde (Sigma-Aldrich), permeabilized with Triton X-100 (0.5% (v/v) in PBS) and blocked with bovine serum albumin (BSA, 5% (w/v) in PBS). Cells were next incubated with primary antibodies HSP47 (ab54874, Abcam) and PDI (ab3672, Abcam) and secondary antibodies (Alexa Fluor 488 conjugated goat-anti-mouse (1:1500, Molecular Probes, Life Technologies, Carlsbad, CA, USA) and Alexa Fluor 594 conjugated donkey-anti-rabbit (1:1500, Molecular Probes, Life Technologies) diluted in 2% BSA/PBS. Nuclei were counterstained with DAPI (4’-6’-diamidino-2-phenylinodole hydroxychloride, Vector Laboratories, Burlingame, CA, USA). Stained preparations were analyzed using the Axio Observer.Z1 fluorescence microscope (Carl Zeiss Microscopy, Thornwood, NY, USA). Images were captured with the Zen pro software and processed with Fiji. [41]

### Expression and purification of recombinant HSP47

Wild-type HSP47 was expressed and purified as described previously. [42] Shortly, a strep-tagged construct containing the canine HSP47 sequence (_36_LSP… RDEL_418_) in a pJExpress411 vector was transformed into the BL21 (DE3) *Escherichia coli* strain, the protein expressed at 37°C for 4h and purified using a Streptactin column. The protein was polished using size-exclusion chromatography (Superdex 200) in 50 mM Hepes (pH 7.5), 150 mM NaCl, 2 mM DTT and stored aliquoted at -80°C. The R222S variant was introduced using a modified Quikchange mutagenesis protocol [42] and purified as wild-type HSP47.

### HSP47 melting temperature analysis

Protein melting points were determined by differential scanning fluorimetry using both the thermal shift assay and intrinsic tryptophan fluorescence. Thermal shift assay was performed essentially as described earlier. [43] Briefly, a 50 μl solution containing 1 mg/ml protein in 50 mM Tris, 150 mM NaCl, pH 7.5 (TBS) and 1x SYPRO-Orange (Invitrogen, Carlsbad, CA, USA) was heated in a thermocycler (CFX98, BioRad) from 20°C to 95°C with an approximate heating rate of 1°C/min. Fluorescent readings were taken every 0.5°C using the FRET channel (Ex 450–490, Em 560–580) of the device. T_m_ was defined as the minima of the first derivative. All data were measured in triplicates. Intrinsic tryptophan fluorescence was measured using a Prometheus NanoDSF (NanoTemper Technologies GmbH, München, Germany) according to the manufacturer protocol. Briefly, protein in MacIlvaine buffer (0.2 M Na_2_HPO_4_/0.1 M citric acid pH 7.5) was loaded into capillaries and the temperature was increased by 1°C/min from 20°C to 95°C. Intrinsic tryptophan fluorescence was measured at 330 nm and 350 nm and plotted as 350/300 nm ratio. T_m_ was defined as the minima of the first derivative. All data were measured in duplicates. Analysis of the data was performed using the Origin2018b software.

### Biolayer interferometry (BLI)

Measurements of the binding kinetics using biolayer interferometry was performed as described earlier. [44]. Shortly, a foldon stabilized collagen model peptide, containing a (GPP)_5_GPR(GPP)_6_ collagen sequence (GPR-foldon), was biotinylated and immobilized to streptavidin-coupled biosensors (FortéBio, Fremont, CA, USA). Sensors were blocked with 10 μM biocytin and unspecific interactions reduced with multiple load/regeneration cycles using McIlvaine buffer at pH 6 for regeneration. All interaction studies were performed in 50 mM Tris, 150 mM NaCl, pH 7.5 (TBS). All datasets were measured twice. The k_off_ and k_on_ value were individually fitted in Origin 2018b using a 1:1 Langmuir binding model. K_D_ values were calculated by k_off_/k_on_ and the error was calculated based on the rules of propagation of uncertainty.

ΔKD=KD(Δkonkon)2+(Δkoffkoff)2 (1)

### Surface Plasmon Resonance (SPR) analysis

SPR experiments were carried out using a BIAcore X instrument (GE Healthcare, Chicago, IL, USA). Purified type I collagen from mouse tail tendon was immobilized on a CM5 sensor chip by amide coupling. The approximate coupled protein concentration was 6 ng/mm^2^ (6,000 response units) of type I collagen. The experiments were performed at 20°C in HBS-P (10 mM Hepes, pH 7.4, containing 150 mM NaCl and 0.005% Surfactant P20) using a flow rate of 10 μl/min. All curves are the average of at least three replicates and three independent measurements were performed. For the analysis of the binding affinity, the curves were fitted with the Langmuir binding model (BIAevaluation software; GE Healthcare).

### Enzyme-linked immunosorbent assay (ELISA)

For ELISA binding assays, type I collagen from rat tail (BD Bioscience, San Jose, CA, USA) was diluted in TBS and coated at 10 μg/mL (500 ng per well) overnight at room temperature onto 96-well plates (MaxiSorp, Nunc, Roskilde, Denmark). After washing with TBS/0.05% Tween-20, plates were blocked for 1 h at room temperature with 5% milk powder in TBS. His-tagged HSP47 proteins were added at concentrations ranging from 1.2 to 5,000 nM. After washing with TBS/0.05% Tween-20, bound ligands were detected with a HRP-coupled anti-His tag antibody (Miltenyi Biotech, Bergisch Gladbach, Germany) and visualized with tetramethylbenzidine as substrate. The reaction was stopped by adding 10% H_2_SO_4_, and absorption was measured at 450 nm. The data were analyzed using Origin 2018b and fitted using a four-parameter logistic model. [45] All measurements were performed twice and representative curves are shown.

### Biochemical collagen analyses

For steady-state (pro)collagen analyses, fibroblasts were plated in petri dishes and grown in medium (composition as described above) supplemented with 25 μg/ml ascorbic acid for 24 h. Next, confluent cell layers were labeled, and procollagen was harvested, precipitated, digested, and separated as described before. [46]

### Collagen thermal stability analysis

Collagen thermal stability was determined as described in [47]. This analysis was performed in duplicate.

### Circular dichroism measurements

Patient and control dermal fibroblasts were cultured in DMEM with 4.5 g/L glucose, L-glutamine, and sodium pyruvate containing 10% (v/v) FBS (Atlanta Biologicals, Flowery Branch, GA, USA), Pen Strep Glutamine (100×; Life Technology), and 5 mM Hepes in presence of ascorbic acid phosphate (100 μg/mL; Wako Chemicals GmbH, Neuss, Germany). Type I collagen was extracted from conditioned serum-free media of cultured dermal fibroblasts following standard ammonium sulfate precipitation, followed by pepsin digestion in acetic acid and two precipitation steps with 0.7 M NaCl in acetic acid and 2.5 M NaCl at pH7. Circular dichroism spectra were recorded on an AVIV 202 spectropolarimeter (AVIV Biomedical, Inc., Lakewood, NJ) using a Peltier thermostated cell holder and a 1 mm path length rectangular quartz cell (Starna Cells Inc., Atascadero, CA). Protein concentrations were determined by amino acid analysis. The thermal unfolding of collagen molecules was monitored at 222 nm wavelength at -6°C/hour and 1°C/min or 0.1°C/min heating rate. The proteins were measured in 100 mM acetic acid.

### Intracellular folding rate assay

Patient and control fibroblasts were plated in 15 cm dishes and grown to confluency. Following stimulation of procollagen biosynthesis with 50 μg/ml ascorbic acid 2-phosphate for one day, cells were preincubated in pulse medium (Met- and Cys-free DMEM with 10% FBS and 50 μg/ml ascorbic acid 2-phosphate) without L-azidohomoalanine (AHA, Click Chemistry Tools) at 37°C for 30 min to deplete methionine. Pulse labeling was performed with 1 mM AHA in pulse medium at 37°C for 15 min, and cells were incubated in chase media (standard DMEM with 10% FBS, cold methionine and 50 μg/ml ascorbic acid 2-phosphate) at room temperature for different time intervals (*i*.*e*. 2 min, 5 min, 10 min, 15 min, 20 min, 25 min, 30 min and 40 min). After the chase, the cells was washed with PBS, and 1250 μL of M-PER cell lysis buffer (Thermo Scientific) containing a final concentration of 1% NP40 (Thermo Scientific), 200 mg/mL chymotrypsin (Sigma), 80 mg/mL trypsin (Sigma), and 0.5 mg/mL DNASE I (ROCHE) was added directly to the cells. After 30 sec incubation, the cell lysate was collected by a cell scraper into a microcentrifuge tube and centrifuged at the highest speed by a tabletop centrifuge at room temperature for 2 min. The supernatant was transferred to a new 2.0 mL microcentrifuge tube and mixed with 250 μL of 1 M acetic acid containing 0.6 mg/mL pepsin (Sigma) at room temperature for 1 hour. After pepsin digestion, 500 μL of 5 M NaCl was added to the tube and incubated at 4°C for overnight. Type I collagen was precipitated at the highest speed by a tabletop centrifuge. The pellet containing type I collagen was dissolved by 43 μL of TBS and labeled with 2 μL of Click-iT Alexa Fluor 488 sDIBO Alkyne (Thermo Scientific) at room temperature for an hour. This labeled solution was mixed with 15 μL of 4x Bolt^™^ LDS Sample Buffer without reducing agent and heat and separated on precast Bolt^™^ 4–12% Bis-Tris Plus Gels with MES running buffer (Thermo Scientific). The signals from AHA-Alexa Fluor 488 were detected by the ChemiDoc MP imaging system (BioRad) using the software Image Lab version 4.0.1 (BioRad). The intensities of fluorescent signals were measured by Image J. The AHA incorporated type I procollagen as a loading control was purified from cell culture media after the overnight chase with the method as described above. To quantitate the amount of newly synthesized AHA incorporated type I collagen at each chase time point, the fluorescent signal of AHA incorporated type I collagen was normalized to the signal corresponding to the total amount of type I collagen (with and without AHA) stained with GelCode Blue Stain Reagent (Thermo Scientific). This assay was performed 4 times and representative images are shown.

### Secretion rate assay

Patient and control fibroblasts were grown to confluency as described in the ‘*Circular dichroism measurements*’ section, trypsinized, and counted. Cells were adjusted to equalize cell number across different cell types and then resuspended in serum-free medium and recounted. Cells were preincubated in labeling medium (methionine-free Dulbecco’s modified Eagle’s medium without fetal bovine serum but with ascorbate at a final concentration of 50 μg/ml at 37°C with gentle shaking for at least 20 min). Cells were briefly centrifuged, and medium was removed and replaced with 10 ml of labeling medium containing 1 mCi [^35^S]methionine (American Radiolabeled Chemicals, Inc., St. Louis, MO, USA) and incubated with shaking at 37°C for 30 min. Cells were then spun out briefly and resuspended in chase medium (standard Dulbecco’s modified Eagle’s medium with cold methionine and ascorbate) and aliquoted for separate time points (*i*.*e*. 10 min, 20 min, 30 min, 45 min, 60 min, 90 min, 2 h, 3 h and 4 h). Cells were incubated at 37°C with shaking, removed at each time point, and spun out at high speed. Medium was transferred to a labeled fresh tube, and cell pellet and medium were frozen. After thawing 1 ml of lysis buffer (1% Nonidet P-40 and 10 mM EDTA in phosphate-buffered saline) was added to each cell pellet and mixed. Cell lysates and medium were precipitated with ammonium sulfate (200 mg per 1 ml of volume was added to each tube, incubated 2–3 h with shaking at 4°C). Precipitates were centrifuged at high speed for 20–30 min at 4°C and the resultant pellet was resuspended in 0.5 M acetic acid with 0.4 mg/ml pepsin. Samples were digested overnight at 4°C with shaking. Pepsinized collagen extracts were precipitated with 0.7 M NaCl final concentration at 4°C and spun out at high speed for 30 min. Pellets were resuspended in 0.5 M acetic acid and SDS-PAGE sample buffer, run on 6–8% acrylamide gels, dried, and exposed to film for autoradiography. A similar protocol was performed in duplicate using ^14^C-proline and alternative time points (*i*.*e*. 0 min, 20 min, 40 min, 60 min and 120 min).

### Amino acid analyses

Acid- and base-hydrolyzed collagen was analyzed as reported previously. [48] Acid hydrolysis was performed in triplicate and base hydrolysis once.

### Collagen digestion and mass spectrometry analysis

To digest type I collagen in trypsin, lyophilized samples were resuspended in equal volume as the pre-lyophilisate of 1M Tris-HCl pH 8.0 with 20 ng/μL trypsin and digested for 18 hours at 37°C. Identification of peptides produced by proteolytic digest was performed on a Q-TOF Micromass spectrometer (Waters, Billerica, MA, USA) equipped with an electrospray ionization source. Data were collected with the MassLynx (version 4.1) data acquisition software (Waters) and processed using Mascot Distiller (Matrix Software, London, United Kingdom). High performance liquid chromatography was performed with nanoACQUITY (Waters) system using a 75 μm x 100 mm 3 μm Atlantis dC18 column as the analytical column and a 180 μm x 20 mm 5 μm Symmetry C18 column as the trapping column. Chromatographic mobile phases consisted of solvents A (0.1% formic acid and 99.9% water (v/v)) and B (0.1% formic acid and 99.9% acetonitrile (v/v)). Peptide samples were loaded onto the trapping column and equilibrated 4 min in 99% solvent A followed by a 180 min gradient to 60% solvent A, 40% solvent B at a constant flow rate of 0.8 μl/min. Analysis was performed in survey scan mode. Tryptic peptides were identified from MS/MS spectra by a Mascot search against the National Center for Biotechnology Information (NCBI) database (peptide tolerance 1.0 Da, MS/MS tolerance 1.0 Da).

### Protein level analyses by Western blotting

Patient and control dermal fibroblasts were cultured in 60 mm dishes. Cell lysates were extracted using M-PER (Thermo Fisher Scientific) containing Halt^™^ Protease Inhibitor Cocktail, EDTA-Free (Thermo Fisher Scientific) at 4°C. For detection of ATP6, the culture medium was replaced with and without 50 mM DTT after the cells grew to over 80% confluency. After incubation for 90 minutes, the cells were washed twice with PBS and lysates were extracted using RIPA buffer (Thermo Fisher Scientific) containing Halt^™^ Protease Inhibitor Cocktail, EDTA-Free (Thermo Fisher Scientific) at 4°C. After centrifugation, soluble proteins in the extract were mixed with SDS sample buffer with reducing agents. These protein solutions were separated on Bolt 4–12% Bis-Tris Plus Gels (Thermo Fisher Scientific) then transferred to PVDF membranes and Western blots were performed using antibodies listed in S3 Table. Blots were developed with HRP enhanced SuperSignal West Pico Chemiluminescent Substrate (Thermo Fisher Scientific) and detected by ChemiDoc MP imaging system (BioRad) using the software Image Lab version 5.2 (BioRad). The intensities of protein signals were measured by Image J. [41]

### Gelatin sepharose pulldown assay using cell lysate

Patient and control dermal fibroblasts were cultured in 150 mm dishes and cell lysates were prepared with M-PER as described above. Six ml of cell lysates in which the total protein concentration was normalized to 1.0 OD at 280 nm measured in a Cary 4 Series UV-Vis spectrophotometer (Agilent Technologies, Santa Clara, CA, USA) were incubated with 1.5 ml of gelatin Sepharose 4B (GE Healthcare) for 2 h at 4°C. After washing twice with 10 ml TBS buffer followed by a wash with 25 mM Tris buffer, pH 7.4, containing 1 M NaCl and additionally twice with TBS buffer, sepharose beads were eluted with 2X SDS-PAGE sample buffer containing β-mercaptoethanol by heating to 95°C for 5 min. The eluted proteins were separated by SDS-PAGE followed by transfer to PVDF membrane and Western blotting and analyses were performed as described earlier. Gels were also stained with SilverXpress Silver Staining Kit (Thermo Fisher Scientific).

### Statistical analyses

For comparisons between patient and control, we performed unpaired t test to determine whether differences were significant using ORIGIN Pro ver. 9.1 (OriginLab Corp., Northampton, MA, USA) or GraphPad Prism software (v5; GraphPad Software, LaJolla, CA, USA). A *P* value of less than 0.05 was considered statistically significant.

## Supporting information

S1 Fig Melting temperature analysis. Differential Scanning Fluorimetry based on intrinsic tryptophan fluorescence **(A)** and SYPRO Orange binding **(B)** showed a similar melting point for both wild-type (WT) (59.35°C and 54.50°C) and R222S (57.48°C and 54.00°C) protein, indicating that the variant does not cause structural instability.
(TIFF)

S2 Fig Intracellular folding rate assay of type I collagen produced by dermal fibroblast cultures. **(A)** Quantification of the folding rate of type I collagen (n = 4) presented in Fig 4B of the manuscript. To quantitate the amount of newly synthesized AHA incorporated type I collagen at each chase time point, the fluorescent signal of AHA incorporated type I collagen was normalized to the signal corresponding to the total amount of type I collagen (with and without AHA) stained with GelCode Blue Stain Reagent (Thermo Scientific). The signal at 40 minutes of AHA incorporated type I collagen was set to 1 to compare with that of the other chase time points. **(B)** Calculation of the area under the curve (AUC). Each dot represents a separate experiment. Results are shown as mean ± SEM. ns: not significant by unpaired t test. Although the folding appears slightly faster in patient versus control fibroblast, no significant difference could be observed. C: control, P: patient.
(TIFF)

S3 Fig Secretion rate assay of type I collagen produced by dermal fibroblast cultures. **(A)** Quantification of the secretion rate of type I collagen presented in Fig 4C of the manuscript. **(B)** Additional secretion rate assay of type I collagen performed using an alternative experimental setup. **(C)** Quantification of the secretion rate of type I collagen in the medium using the alternative experimental setup (n = 2). Data presented are means ± SEM. Both setups show a near-normal secretion rate for patient compared to control. C: control, P: patient.
(TIFF)

S4 Fig Melting curve analysis of type I collagen. Circular dichroism measurements of type I collagen extracted from control (black) and patient (pink) fibroblasts performed at a heating rate of 1°C/min. C: control, P: patient.
(TIFF)

S5 Fig Amino acid analysis of type I collagen extracted from control and patient dermal fibroblasts. **(A)** Amino acid analysis of type I collagen after acid hydrolysis shows apparently normal levels of prolyl 4-hydroxylation and slightly increased levels of prolyl 3-hydroxylation in patient samples (insert). 3-Hyp: 3-hydroxyproline, 4-Hyp: 4-hydroxyproline, Pro: unmodified proline. **(B)** Amino acid analysis of type I collagen after acid hydrolysis shows increased levels (30%) of lysyl hydroxylation in patient samples. Hyl: hydroxylysine, Lys: unmodified lysine. **(C)** Amino acid analysis of type I collagen after base hydrolysis shows significantly increased glycosylation in patient samples. Glucosyl-galactosyl hydroxylysine content is increased by approximately 50% in patient samples, while no change in galactosyl hydroxylysine is observed. GGH: glucosyl-galactosyl hydroxylysine standard in blue, GH: galactosyl hydroxylysine standard in green. **(D)** Quantification of proline and lysine modifications after acid hydrolysis show significantly increased 3-Hyp, 4-Hyp and Hyl levels in patient versus control samples. Data presented are means ± SEM. *: p<0.05, **: p<0.01, ***: p<0.001, unpaired t test. **(E)** Calculation of the percentage and increase in modification. The percentage was calculated as the number of hydroxylated lysine/proline residues divided by the total number of lysine/proline residues. Mean is represented with SEM between brackets. C: control, P: patient.
(TIFF)

S6 Fig Mass spectrometry data of trypsin digested type I collagen. Type I collagen was extracted from conditioned culture media of control and patient dermal fibroblasts. **(A,B)** 3-Hydroxylation of proline residues is normal at the A1 site (P986) **(A)** and increased at the A3 site (P707) **(B)** of the type I collagen α1-chain. **(C-D)** Additional sugar attachment is observed at lysyl hydroxylation sites at residues K174 **(C)** and K531 **(D)** of the type I collagen α1-chain from patient cells. **(E)** Increased lysyl hydroxylation at residue K756 of the type I collagen α1-chain from patient cells. The peptide sequence is shown above the mass spectra and the examined residue is depicted in red. C: control, P: patient.
(TIFF)

S7 Fig Western blots against proteins of the molecular ensemble involved in collagen biosynthesis using cell lysates. The cell lysate extracted from patient and control dermal fibroblast cultures were electrophoresed on a Bolt 4–12% Bis-Tris Plus Gel followed by transfer to PVDF and Western blotting. Antibodies used to the blotting are listed in S3 Table. The arrowhead indicates the migration corresponding to the molecular weight of the protein, which was used for further quantitative analysis. C: control, P: patient.
(TIFF)

S8 Fig Expression analysis of transcripts encoding proteins of the molecular ensemble involved in collagen biosynthesis. RT-qPCR was performed to assess expression levels. Protein names are indicated and the corresponding HUGO-approved gene name is included between brackets. Data presented are means ± SEM and individual data points represent independently prepared total RNA. *: p<0.05, **: p<0.01, ***: p<0.001 and ns: not significant by unpaired t test. C: control, P: patient.
(TIFF)

S9 Fig Western blots against proteins of the molecular ensemble involved in collagen biosynthesis that bind gelatin. Western blots for the gelatin sepharose elution fraction of proteins that **(A)** bind to gelatin and **(B)** do not bind gelatin. The cell lysate extracted from control and patient dermal fibroblasts and the eluted fractions from gelatin sepharose are depicted. The red color on the gel bands indicates that a membrane was overexposed and the signals were saturated. The asterisk (*) in the LH3 blot highlights the predicted migration of LH3. The additional, non-specific bands in the input lysate of the LH3 blot, compared to S7 Fig, could be due to much higher protein concentration of cell lysate for the gelatin sepharose binding experiments. Antibodies used for the blots are listed in S3 Table. ‘-‘ indicates the eluted fractions are from gelatin sepharose mixed with TBS buffer instead of cell lysate as a blank. C: control, P: patient.
(TIFF)

S10 Fig Evaluation of ER stress and autophagy signatures in dermal fibroblasts. **(A)** Cells were treated with tunicamycin (+Tu), thapsigargin (+Th) or left untreated (-) as indicated and evaluated for the presence of BiP, XBP1 spliced and CHOP. **(B)** Cells were either treated with DTT (+) or left untreated (-) as indicated. ATF6 (N) and ATF6 (C) indicate the predicted size of native full length and cleaved form by ER stress, respectively. ATF6 (C) is overlapping with a non-specific protein. The black line spacing denotes irrelevant lanes that were eliminated from the image. **(C)** Endogenous levels of eIF2α and phospho-eIF2α were evaluated. **(D)** Endogenous levels of the autophagy marker LC3 were evaluated. LC3-I and LC3-II represent the cytosolic form and the autophagosome-bound form of LC3, respectively. β-tubulin was used as loading control. C: control, P: patient.
(TIFF)

S11 Fig Mapping of the amino acid residues with currently identified missense variants in HSP47. Crystal structure of the HSP47-collagen interaction with the collagen trimer depicted centrally, surrounded by two HSP47 molecules depicted in light and dark grey. Missense variants identified in humans and dog (with asterisk) are indicated in different colors on the HSP47 crystal structure. While residues L50, L78, L326 (canine, with asterisk) and R405 are located further away from the collagen interaction surface, R222 (present study) and M237 are located close to the interaction surface.
(TIFF)

S1 Table Clinical summary of individuals with pathogenic *SERPINH1* variants identified so far. NA: not assessed, ^1^ personal communication with the authors.
(XLSX)

S2 Table *In silico* predictions and reported allele frequency for the identified p.(Arg222Ser) substitution. (XLSX)

S3 Table Antibodies used for Western blot analyses. (XLSX)

## References

[1] Birk DE, Bruckner P. Collagens, Suprastructures, and Collagen Fibril Assembly The Extracellular Matrix: an Overview. Berlin, Heidelberg: Springer Berlin Heidelberg; 2010 pp. 77–115. 10.1007/978-3-642-16555-9_3

[2] Ishikawa Y, Bächinger HP. A molecular ensemble in the rER for procollagen maturation. Biochim Biophys Acta. 2013;1833: 2479–2491. 10.1016/j.bbamcr.2013.04.008 23602968

[3] Nagata K, Saga S, Yamada KM. A major collagen-binding protein of chick embryo fibroblasts is a novel heat shock protein. J Cell Biol. The Rockefeller University Press; 1986;103: 223–229. 10.1083/jcb.103.1.223 PMC2113802 3722264

[4] Nagata K. Hsp47: a collagen-specific molecular chaperone. Trends Biochem Sci. 1996;21: 22–26. 10.1016/0968-0004(96)80881-4 8848834

[5] Nagata K. Expression and function of heat shock protein 47: a collagen-specific molecular chaperone in the endoplasmic reticulum. Matrix Biol. 1998;16: 379–386. 10.1016/s0945-053x(98)90011-7 9524358

[6] Ono T, Miyazaki T, Ishida Y, Uehata M, Nagata K. Direct in vitro and in vivo evidence for interaction between Hsp47 protein and collagen triple helix. J Biol Chem. American Society for Biochemistry and Molecular Biology; 2012;287: 6810–6818. 10.1074/jbc.M111.280248 22235129 PMC3307285

[7] Koide T, Nishikawa Y, Asada S, Yamazaki CM, Takahara Y, Homma DL, et al Specific recognition of the collagen triple helix by chaperone HSP47. II. The HSP47-binding structural motif in collagens and related proteins. J Biol Chem. American Society for Biochemistry and Molecular Biology; 2006;281: 11177–11185. 10.1074/jbc.M601369200 16484215

[8] Widmer C, Gebauer JM, Brunstein E, Rosenbaum S, Zaucke F, Drögemüller C, et al Molecular basis for the action of the collagen-specific chaperone Hsp47/SERPINH1 and its structure-specific client recognition. Proc Natl Acad Sci USA. National Acad Sciences; 2012;109: 13243–13247. 10.1073/pnas.1208072109 22847422 PMC3421173

[9] Makareeva E, Leikin S. Procollagen triple helix assembly: an unconventional chaperone-assisted folding paradigm. Lu J, editor. PLoS ONE. Public Library of Science; 2007;2: e1029 10.1371/journal.pone.0001029 17925877 PMC2000351

[10] Bonfanti L, Mironov AA, Martínez-Menárguez JA, Martella O, Fusella A, Baldassarre M, et al Procollagen traverses the Golgi stack without leaving the lumen of cisternae: evidence for cisternal maturation. Cell. 1998;95: 993–1003. 10.1016/s0092-8674(00)81723-7 9875853

[11] Ishikawa Y, Ito S, Nagata K, Sakai LY, Bächinger HP. Intracellular mechanisms of molecular recognition and sorting for transport of large extracellular matrix molecules. Proc Natl Acad Sci USA. National Acad Sciences; 2016;113: E6036–E6044. 10.1073/pnas.1609571113 27679847 PMC5068301

[12] Satoh M, Hirayoshi K, Yokota S, Hosokawa N, Nagata K. Intracellular interaction of collagen-specific stress protein HSP47 with newly synthesized procollagen. J Cell Biol. 1996;133: 469–483. 10.1083/jcb.133.2.469 8609177 PMC2120794

[13] Ishida Y, Nagata K. Hsp47 as a collagen-specific molecular chaperone. Meth Enzymol. Elsevier; 2011;499: 167–182. 10.1016/B978-0-12-386471-0.00009-2 21683254

[14] Nagai N, Hosokawa M, Itohara S, Adachi E, Matsushita T, Hosokawa N, et al Embryonic lethality of molecular chaperone hsp47 knockout mice is associated with defects in collagen biosynthesis. J Cell Biol. 2000;150: 1499–1506. 10.1083/jcb.150.6.1499 10995453 PMC2150697

[15] Drögemüller C, Becker D, Brunner A, Haase B, Kircher P, Seeliger F, et al A missense mutation in the SERPINH1 gene in Dachshunds with osteogenesis imperfecta. Barsh GS, editor. PLoS Genet. Public Library of Science; 2009;5: e1000579 10.1371/journal.pgen.1000579 19629171 PMC2708911

[16] Christiansen HE, Schwarze U, Pyott SM, AlSwaid A, Balwi Al M, Alrasheed S, et al Homozygosity for a missense mutation in SERPINH1, which encodes the collagen chaperone protein HSP47, results in severe recessive osteogenesis imperfecta. Am J Hum Genet. 2010;86: 389–398. 10.1016/j.ajhg.2010.01.034 20188343 PMC2833387

[17] Duran I, Nevarez L, Sarukhanov A, Wu S, Lee K, Krejci P, et al HSP47 and FKBP65 cooperate in the synthesis of type I procollagen. Hum Mol Genet. Oxford University Press; 2014;24: 1918–1928. 10.1093/hmg/ddu608 25510505 PMC4355024

[18] Essawi O, Symoens S, Fannana M, Darwish M, Farraj M, Willaert A, et al Genetic analysis of osteogenesis imperfecta in the Palestinian population: molecular screening of 49 affected families. Mol Genet Genomic Med. 2017;86: 551 10.1002/mgg3.331 29150909 PMC5823677

[19] Marshall C, Lopez J, Crookes L, Pollitt RC, Balasubramanian M. A novel homozygous variant in SERPINH1 associated with a severe, lethal presentation of osteogenesis imperfecta with hydranencephaly. Gene. 2016;595: 49–52. 10.1016/j.gene.2016.09.035 27677223

[20] Song Y, Zhao D, Xu X, Lv F, Li L, Jiang Y, et al Novel compound heterozygous mutations in SERPINH1 cause rare autosomal recessive osteogenesis imperfecta type X. Osteoporos Int. Springer London; 2018;387: 1657–8. 10.1007/s00198-018-4448-2 29520608

[21] Schwarze U, Cundy T, Liu YJ, Hofman PL, Byers PH. Compound heterozygosity for a frameshift mutation and an upstream deletion that reduces expression of SERPINH1 in siblings with a moderate form of osteogenesis imperfecta. Am J Med Genet A. John Wiley & Sons, Ltd; 2019;260: 1734 10.1002/ajmg.a.61170 31179625

[22] Barnes AM, Chang W, Morello R, Cabral WA, Weis M, Eyre DR, et al Deficiency of Cartilage-Associated Protein in Recessive Lethal Osteogenesis Imperfecta. N Engl J Med.; 2006;355: 2757–64. 10.1056/NEJMoa063804 17192541 PMC7509984

[23] Cabral WA, Chang W, Barnes AM, Weis M, Scott MA, Leikin S, et al Prolyl 3-hydroxylase 1 Deficiency Causes a Recessive Metabolic Bone Disorder Resembling Lethal/Severe Osteogenesis Imperfecta. Nat Genet. 2007;39: 359–65. 10.1038/ng1968 17277775 PMC7510175

[24] Yamauchi M, Sricholpech M. Lysine post-translational modifications of collagen. Essays Biochem. Portland Press Limited; 2012;52: 113–133. 10.1042/bse0520113 22708567 PMC3499978

[25] Lindert U, Weis MA, Rai J, Seeliger F, Hausser I, Leeb T, et al Molecular Consequences of Defective SERPINH1/HSP47 in the Dachshund Natural Model of Osteogenesis Imperfecta. J Biol Chem. American Society for Biochemistry and Molecular Biology; 2015; 10.1074/jbc.M115.661025 26004778 PMC4505018

[26] Koide T, Takahara Y, Asada S, Nagata K. Xaa-Arg-Gly triplets in the collagen triple helix are dominant binding sites for the molecular chaperone HSP47. J Biol Chem. American Society for Biochemistry and Molecular Biology; 2002;277: 6178–6182. 10.1074/jbc.M106497200 11751879

[27] Koide T, Asada S, Takahara Y, Nishikawa Y, Nagata K, Kitagawa K. Specific recognition of the collagen triple helix by chaperone HSP47: minimal structural requirement and spatial molecular orientation. J Biol Chem. American Society for Biochemistry and Molecular Biology; 2006;281: 3432–3438. 10.1074/jbc.M509707200 16326708

[28] Ito S, Nagata K. Mutants of collagen-specific molecular chaperone Hsp47 causing osteogenesis imperfecta are structurally unstable with weak binding affinity to collagen. Biochem Biophys Res Commun. 2015;469: 437–442 10.1016/j.bbrc.2015.12.028 26692483

[29] Marini JC, Forlino A, Bächinger HP, Bishop NJ, Byers PH, Paepe AD, et al Osteogenesis imperfecta. Nat Rev Dis Primers. Nature Publishing Group; 2017;3: 17052 10.1038/nrdp.2017.52 28820180

[30] Pokidysheva E, Mizuno K, Bächinger HP. The Collagen Folding Machinery: Biosynthesis and Post-Translational Modifications of Collagens. Osteogenesis Imperfecta. 2014 pp. 57–70.

[31] Ishikawa Y, Holden P, Bächinger HP. Heat Shock Protein 47 and 65-kDa FK506-binding Protein Weakly but Synergistically Interact During Collagen Folding in the Endoplasmic Reticulum. J Biol Chem. 2017;292: 17216–17224. 10.1074/jbc.M117.802298 28860186 PMC5655501

[32] Asada S, Koide T, Yasui H, Nagata K. Effect of HSP47 on Prolyl 4-hydroxylation of Collagen Model Peptides. Cell Struct Funct. 1999;24: 187–96. 10.1247/csf.24.187 10532353

[33] Duran I, Martin JH, Weis M, Krejci P, Konik P, Li B, et al A Chaperone Complex Formed by HSP47, FKBP65, and BiP Modulates Telopeptide Lysyl Hydroxylation of Type I Procollagen. J Bone Miner Res. 2017 6;32(6):1309–1319. 10.1002/jbmr.3095 28177155 PMC5466459

[34] Marutani T, Yamamoto A, Nagai N, Kubota H, Nagata K. Accumulation of type IV collagen in dilated ER leads to apoptosis in Hsp47-knockout mouse embryos via induction of CHOP. J Cell Sci. The Company of Biologists Ltd; 2004;117: 5913–5922. 10.1242/jcs.01514 15522896

[35] Ishida Y, Yamamoto A, Kitamura A, Lamandé SR, Yoshimori T, Bateman JF, et al Autophagic elimination of misfolded procollagen aggregates in the endoplasmic reticulum as a means of cell protection. Mol Biol Cell. American Society for Cell Biology; 2009;20: 2744–2754. 10.1091/mbc.e08-11-1092 19357194 PMC2688553

[36] Ishida Y, Kubota H, Yamamoto A, Kitamura A, Bächinger HP, Nagata K. Type I collagen in Hsp47-null cells is aggregated in endoplasmic reticulum and deficient in N-propeptide processing and fibrillogenesis. Mol Biol Cell. American Society for Cell Biology; 2006;17: 2346–2355. 10.1091/mbc.e05-11-1065 16525016 PMC1446091

[37] Forlino A, Cabral WA, Barnes AM, Marini JC. New perspectives on osteogenesis imperfecta. Nat Rev Endocrinol. 2011;7: 540–557. 10.1038/nrendo.2011.81 21670757 PMC3443407

[38] Bettica P, Baylink DJ, Moro L. Galactosyl Hydroxylysine and Deoxypyridinoline: A Methodological Comparison. Eur J Clin Chem Clin Biochem. 1993;31: 459–65. 10.1515/cclm.1993.31.7.459 8399787

[39] De Leeneer K, Hellemans J, Steyaert W, Lefever S, Vereecke I, Debals E, et al Flexible, Scalable and Efficient Targeted Resequencing on a Benchtop Sequencer for Variant Detection in Clinical Practice. Hum Mutat. 2014;36: 379–387. 10.1002/humu.22739 25504618

[40] Richards S, Aziz N, Bale S, Bick D, Das S, Gastier-Foster J, et al Standards and guidelines for the interpretation of sequence variants: a joint consensus recommendation of the American College of Medical Genetics and Genomics and the Association for Molecular Pathology. Springer Nature; 2015 pp. 405–424. 10.1038/gim.2015.30 25741868 PMC4544753

[41] Schindelin J, Arganda-Carreras I, Frise E, Kaynig V, Longair M, Pietzsch T, et al Fiji: an open-source platform for biological-image analysis. Nat Methods. 2012;9: 676–682. 10.1038/nmeth.2019 22743772 PMC3855844

[42] Zheng L., Baumann U., Reymond J.L. An efficient one-step site-directed and site-saturation mutagenesis protocol. Nucleic Acids Research, 2004;32: e115 10.1093/nar/gnh110 15304544 PMC514394

[43] Ericsson U.B., Hallberg B.M., Detitta G.T., Dekker N., Nordlund P. Thermofluor-based high-throughput stability optimization of proteins for structural studies. Analytical Biochemistry; 2006;357: 289–298. 10.1016/j.ab.2006.07.027 16962548

[44] Oecal S, Socher E, Uthoff M, Ernst C, Zaucke F, Sticht H, et al The pH-Dependent Client Release from the Collagen-Specific Chaperone HSP47 is Triggered by a Tandem Histidine Pair. J Biol Chem. American Society for Biochemistry and Molecular Biology; 2016;291: 12612–12626. 10.1074/jbc.M115.706069 27129216 PMC4933464

[45] Findlay JWA, Dillard RF. Appropriate calibration curve fitting in ligand binding assays. AAPS J. Springer-Verlag; 2007;9: E260–7. 10.1208/aapsj0902029 17907767 PMC2751416

[46] Syx D, De Wandele I, Symoens S, De Rycke R, Hougrand O, Voermans N, et al Bi-allelic AEBP1 mutations in two patients with Ehlers-Danlos syndrome. Hum Mol Genet. 2019;28: 1853–1864. 10.1093/hmg/ddz024 30668708

[47] Pace JM, Kuslich CD, Willing MC, Byers PH. Disruption of one intra-chain disulphide bond in the carboxyl-terminal propeptide of the proalpha1(I) chain of type I procollagen permits slow assembly and secretion of overmodified, but stable procollagen trimers and results in mild osteogenesis imperfecta. 2001;38: 443–449. 10.1136/jmg.38.7.443 PMC1757177 11432962

[48] Vranka JA, Pokidysheva E, Hayashi L, Zientek K, Mizuno K, Ishikawa Y, et al Prolyl 3-hydroxylase 1 null mice display abnormalities in fibrillar collagen-rich tissues such as tendons, skin, and bones. J Biol Chem. American Society for Biochemistry and Molecular Biology; 2010;285: 17253–17262. 10.1074/jbc.M110.102228 20363744 PMC2878055

