# Supplementary figures and images for: Aberrant binding of mutant HSP47 affects posttranslational modification of type I collagen and leads to osteogenesis imperfecta
Source: PLoS Genet. 2021 Feb 1;17(2):e1009339. doi: 10.1371/journal.pgen.1009339 (PMC7877763; doi:10.1371/journal.pgen.1009339)

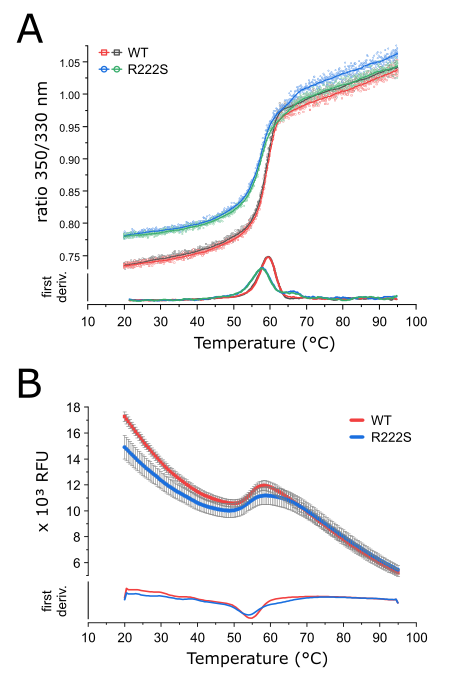

Supplement: S1 Fig — Differential Scanning Fluorimetry based on intrinsic tryptophan fluorescence (A) and SYPRO Orange binding (B) showed a similar melting point for both wild-type (WT) (59.35°C and 54.50°C) and R222S (57.48°C and 54.00°C) protein, indicating that the variant does not cause structural instability. (TIFF) [file pgen.1009339.s001.tiff]

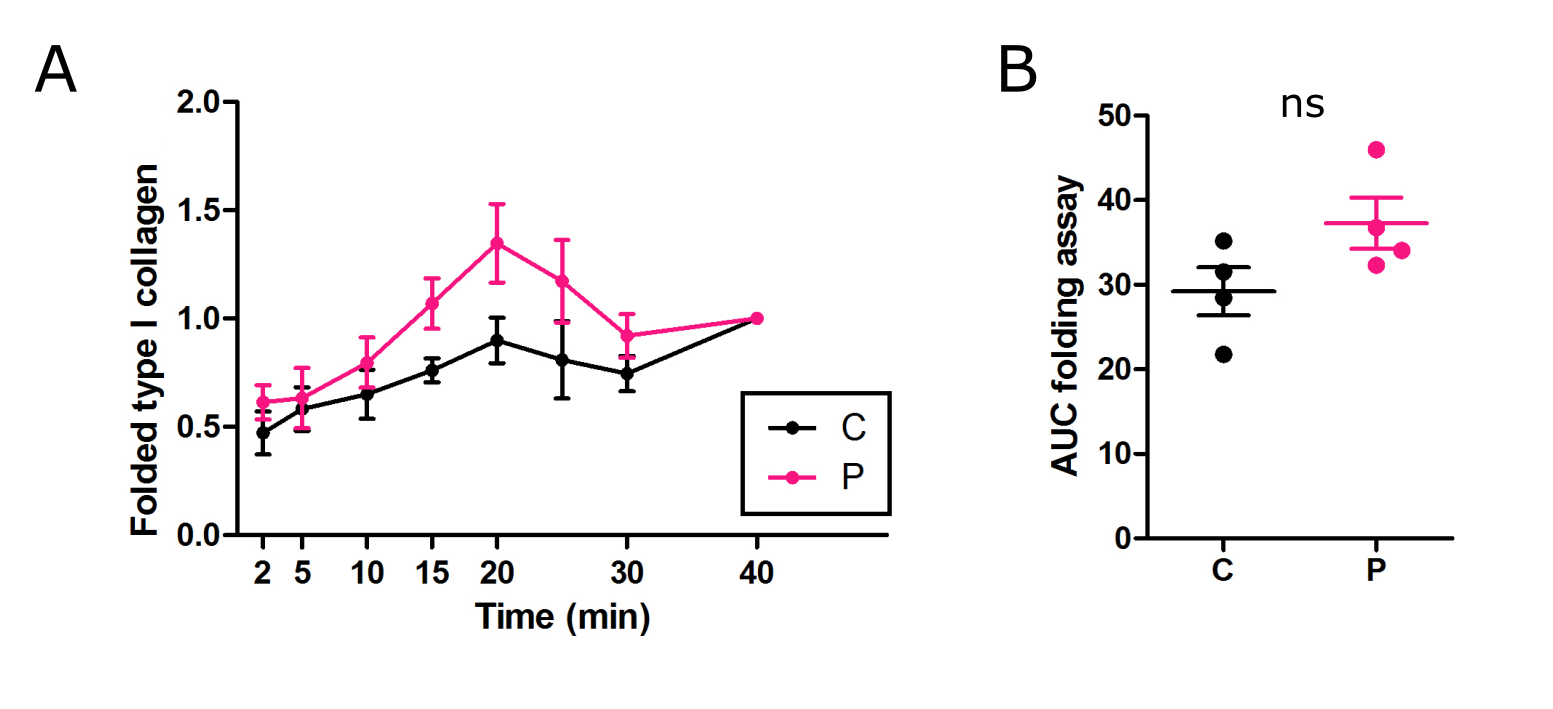

Supplement: S2 Fig — (A) Quantification of the folding rate of type I collagen (n = 4) presented in Fig 4B of the manuscript. To quantitate the amount of newly synthesized AHA incorporated type I collagen at each chase time point, the fluorescent signal of AHA incorporated type I collagen was normalized to the signal corresponding to the total amount of type I collagen (with and without AHA) stained with GelCode Blue Stain Reagent (Thermo Scientific). The signal at 40 minutes of AHA incorporated type I collagen was set to 1 to compare with that of the other chase time points. (B) Calculation of the area under the curve (AUC). Each dot represents a separate experiment. Results are shown as mean ± SEM. ns: not significant by unpaired t test. Although the folding appears slightly faster in patient versus control fibroblast, no significant difference could be observed. C: control, P: patient. (TIFF) [file pgen.1009339.s002.tiff]

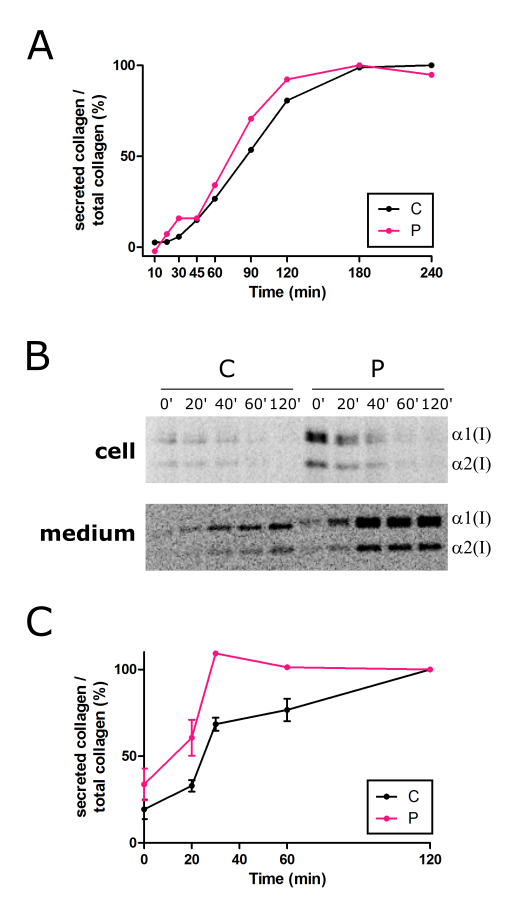

Supplement: S3 Fig — (A) Quantification of the secretion rate of type I collagen presented in Fig 4C of the manuscript. (B) Additional secretion rate assay of type I collagen performed using an alternative experimental setup. (C) Quantification of the secretion rate of type I collagen in the medium using the alternative experimental setup (n = 2). Data presented are means ± SEM. Both setups show a near-normal secretion rate for patient compared to control. C: control, P: patient. (TIFF) [file pgen.1009339.s003.tiff]

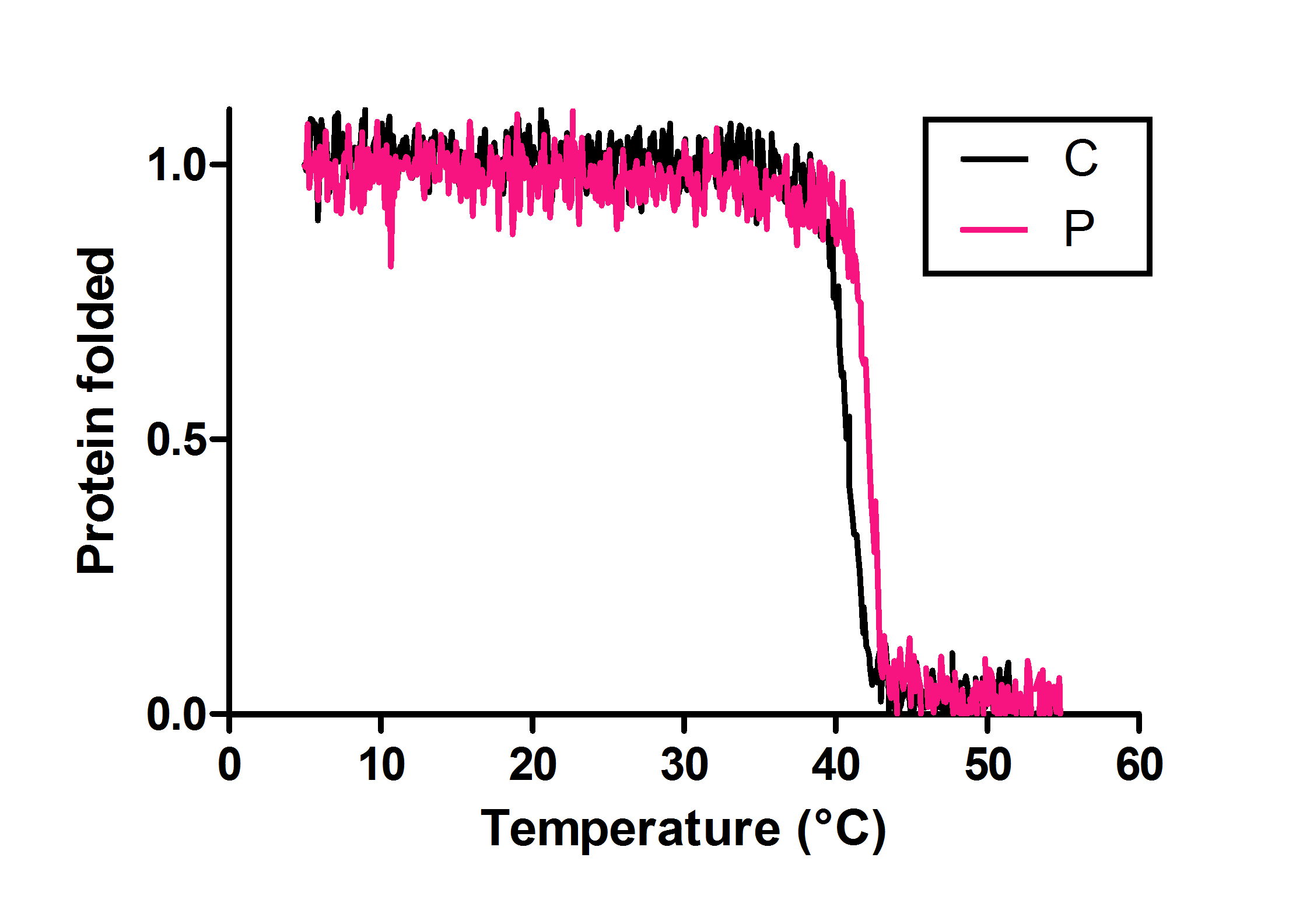

Supplement: S4 Fig — Circular dichroism measurements of type I collagen extracted from control (black) and patient (pink) fibroblasts performed at a heating rate of 1°C/min. C: control, P: patient. (TIFF) [file pgen.1009339.s004.tiff]

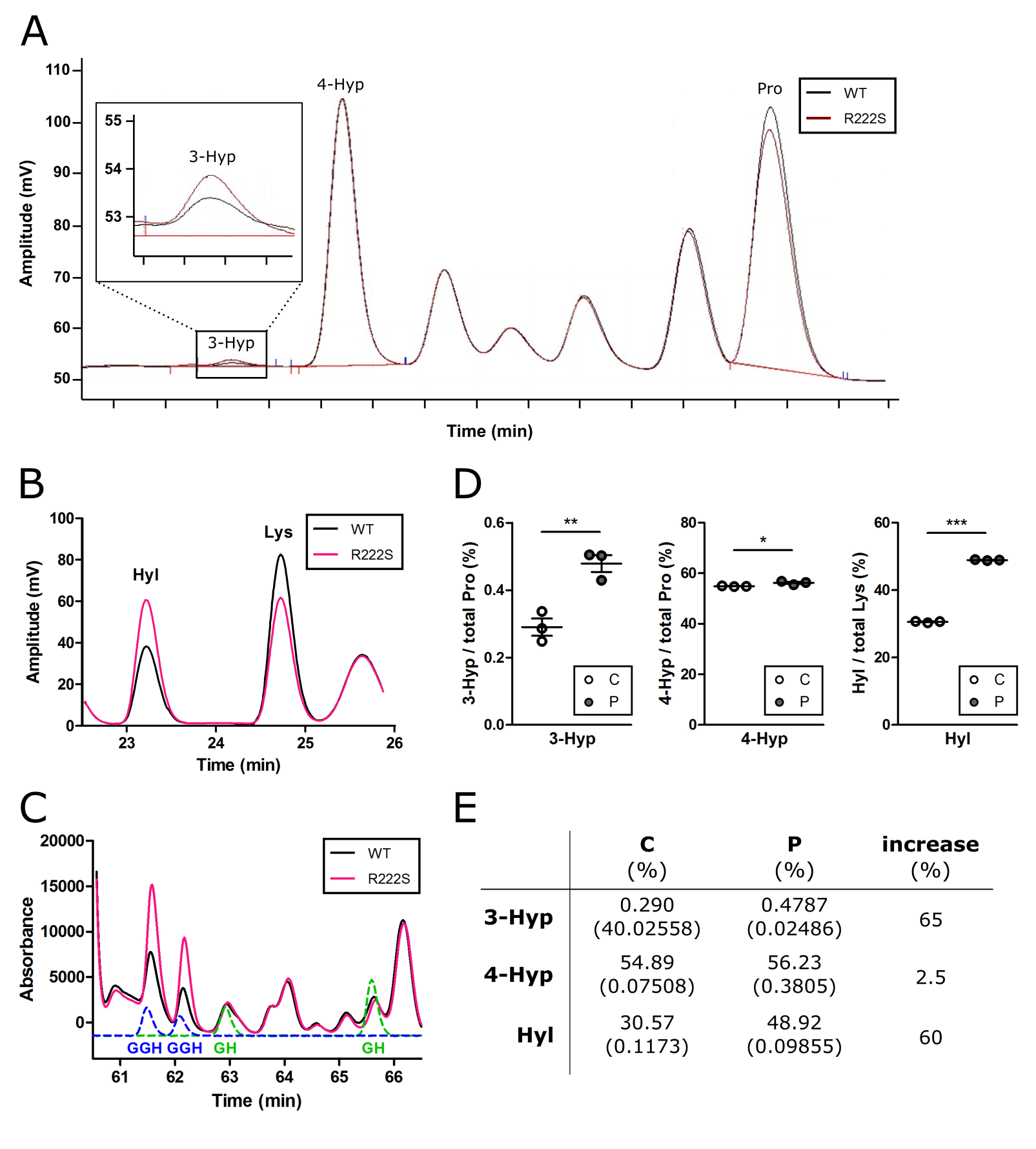

Supplement: S5 Fig — (A) Amino acid analysis of type I collagen after acid hydrolysis shows apparently normal levels of prolyl 4-hydroxylation and slightly increased levels of prolyl 3-hydroxylation in patient samples (insert). 3-Hyp: 3-hydroxyproline, 4-Hyp: 4-hydroxyproline, Pro: unmodified proline. (B) Amino acid analysis of type I collagen after acid hydrolysis shows increased levels (30%) of lysyl hydroxylation in patient samples. Hyl: hydroxylysine, Lys: unmodified lysine. (C) Amino acid analysis of type I collagen after base hydrolysis shows significantly increased glycosylation in patient samples. Glucosyl-galactosyl hydroxylysine content is increased by approximately 50% in patient samples, while no change in galactosyl hydroxylysine is observed. GGH: glucosyl-galactosyl hydroxylysine standard in blue, GH: galactosyl hydroxylysine standard in green. (D) Quantification of proline and lysine modifications after acid hydrolysis show significantly increased 3-Hyp, 4-Hyp and Hyl levels in patient versus control samples. Data presented are means ± SEM. *: p<0.05, **: p<0.01, ***: p<0.001, unpaired t test. (E) Calculation of the percentage and increase in modification. The percentage was calculated as the number of hydroxylated lysine/proline residues divided by the total number of lysine/proline residues. Mean is represented with SEM between brackets. C: control, P: patient. (TIFF) [file pgen.1009339.s005.tiff]

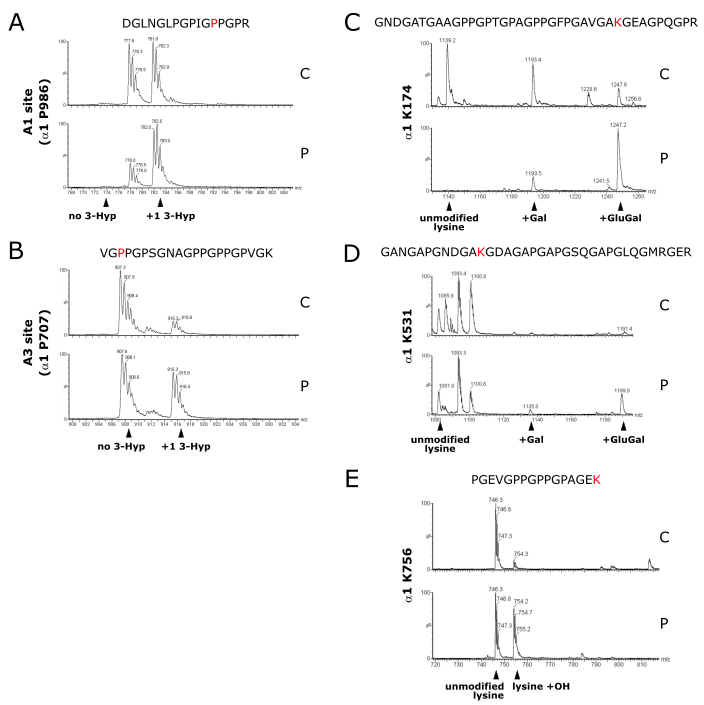

Supplement: S6 Fig — Type I collagen was extracted from conditioned culture media of control and patient dermal fibroblasts. (A,B) 3-Hydroxylation of proline residues is normal at the A1 site (P986) (A) and increased at the A3 site (P707) (B) of the type I collagen α1-chain. (C-D) Additional sugar attachment is observed at lysyl hydroxylation sites at residues K174 (C) and K531 (D) of the type I collagen α1-chain from patient cells. (E) Increased lysyl hydroxylation at residue K756 of the type I collagen α1-chain from patient cells. The peptide sequence is shown above the mass spectra and the examined residue is depicted in red. C: control, P: patient. (TIFF) [file pgen.1009339.s006.tiff]

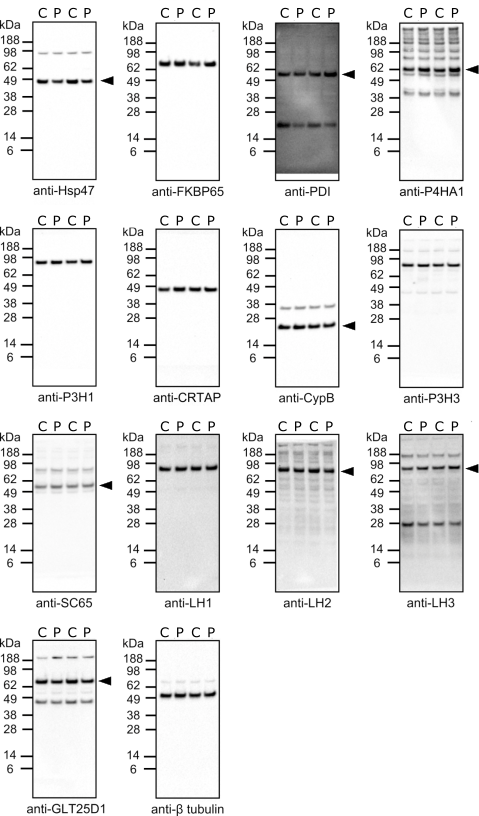

Supplement: S7 Fig — The cell lysate extracted from patient and control dermal fibroblast cultures were electrophoresed on a Bolt 4–12% Bis-Tris Plus Gel followed by transfer to PVDF and Western blotting. Antibodies used to the blotting are listed in S3 Table. The arrowhead indicates the migration corresponding to the molecular weight of the protein, which was used for further quantitative analysis. C: control, P: patient. (TIFF) [file pgen.1009339.s007.tiff]

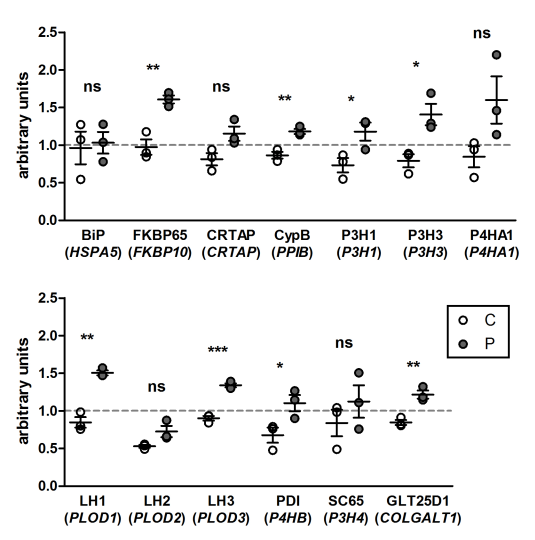

Supplement: S8 Fig — RT-qPCR was performed to assess expression levels. Protein names are indicated and the corresponding HUGO-approved gene name is included between brackets. Data presented are means ± SEM and individual data points represent independently prepared total RNA. *: p<0.05, **: p<0.01, ***: p<0.001 and ns: not significant by unpaired t test. C: control, P: patient. (TIFF) [file pgen.1009339.s008.tiff]

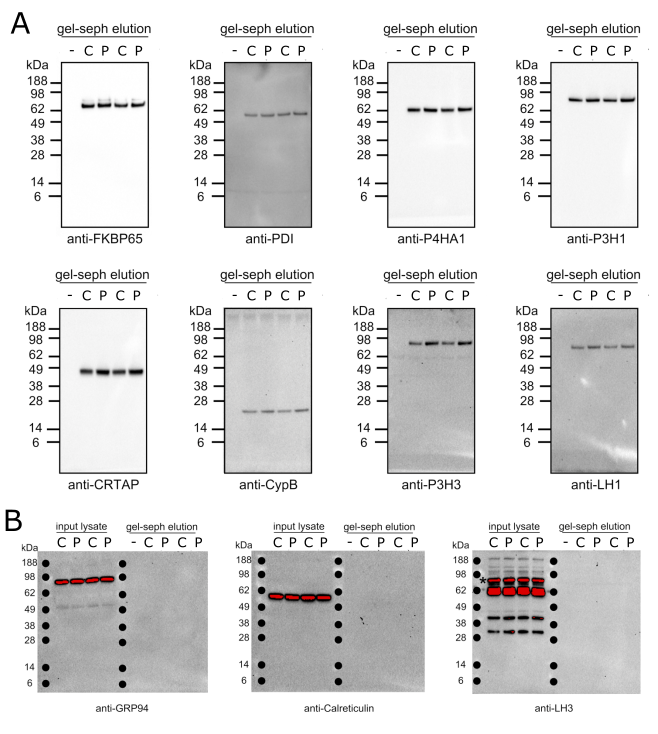

Supplement: S9 Fig — Western blots for the gelatin sepharose elution fraction of proteins that (A) bind to gelatin and (B) do not bind gelatin. The cell lysate extracted from control and patient dermal fibroblasts and the eluted fractions from gelatin sepharose are depicted. The red color on the gel bands indicates that a membrane was overexposed and the signals were saturated. The asterisk (*) in the LH3 blot highlights the predicted migration of LH3. The additional, non-specific bands in the input lysate of the LH3 blot, compared to S7 Fig, could be due to much higher protein concentration of cell lysate for the gelatin sepharose binding experiments. Antibodies used for the blots are listed in S3 Table. ‘-‘ indicates the eluted fractions are from gelatin sepharose mixed with TBS buffer instead of cell lysate as a blank. C: control, P: patient. (TIFF) [file pgen.1009339.s009.tiff]

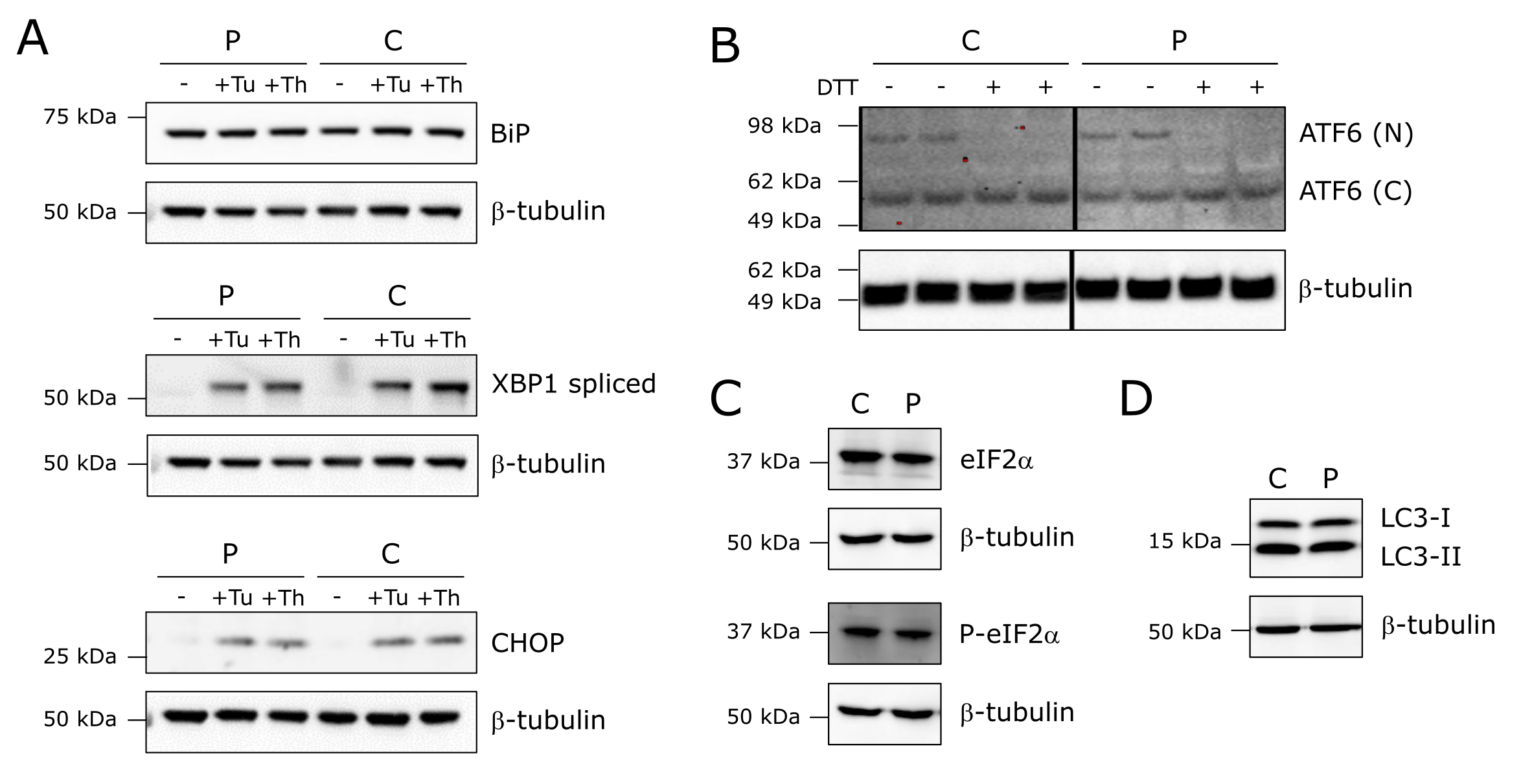

Supplement: S10 Fig — (A) Cells were treated with tunicamycin (+Tu), thapsigargin (+Th) or left untreated (-) as indicated and evaluated for the presence of BiP, XBP1 spliced and CHOP. (B) Cells were either treated with DTT (+) or left untreated (-) as indicated. ATF6 (N) and ATF6 (C) indicate the predicted size of native full length and cleaved form by ER stress, respectively. ATF6 (C) is overlapping with a non-specific protein. The black line spacing denotes irrelevant lanes that were eliminated from the image. (C) Endogenous levels of eIF2α and phospho-eIF2α were evaluated. (D) Endogenous levels of the autophagy marker LC3 were evaluated. LC3-I and LC3-II represent the cytosolic form and the autophagosome-bound form of LC3, respectively. β-tubulin was used as loading control. C: control, P: patient. (TIFF) [file pgen.1009339.s010.tiff]

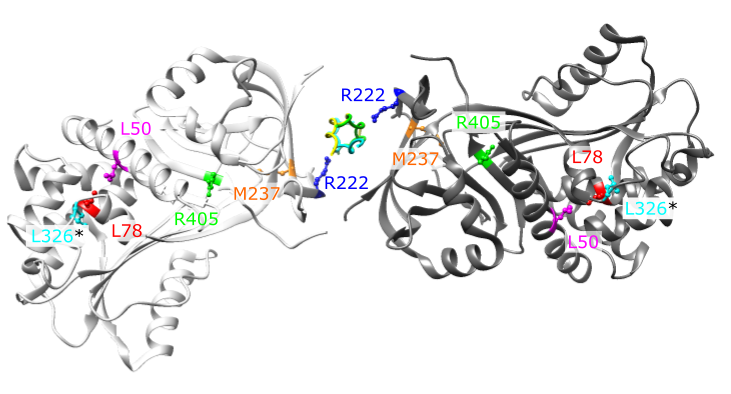

Supplement: S11 Fig — Crystal structure of the HSP47-collagen interaction with the collagen trimer depicted centrally, surrounded by two HSP47 molecules depicted in light and dark grey. Missense variants identified in humans and dog (with asterisk) are indicated in different colors on the HSP47 crystal structure. While residues L50, L78, L326 (canine, with asterisk) and R405 are located further away from the collagen interaction surface, R222 (present study) and M237 are located close to the interaction surface. (TIFF) [file pgen.1009339.s011.tiff]
